# TRIP13 promotes metastasis of colorectal cancer regardless of p53 and microsatellite instability status

**DOI:** 10.1002/1878-0261.12821

**Published:** 2020-10-28

**Authors:** Sumit Agarwal, Michael Behring, Hyung‐Gyoon Kim, Darshan S. Chandrashekar, Balabhadrapatruni V. S. K. Chakravarthi, Nirzari Gupta, Prachi Bajpai, Amr Elkholy, Sameer Al Diffalha, Pran K. Datta, Martin J. Heslin, Sooryanarayana Varambally, Upender Manne

**Affiliations:** ^1^ Department of Pathology University of Alabama at Birmingham AL USA; ^2^ Department of Chemistry University of Alabama at Birmingham AL USA; ^3^ Division of Hematology and Oncology Department of Medicine University of Alabama at Birmingham AL USA; ^4^ Department of Surgery University of Alabama at Birmingham AL USA; ^5^ O'Neal Comprehensive Cancer Center University of Alabama at Birmingham AL USA

**Keywords:** colorectal cancer, EGFR, FGFR4, metastasis, TRIP13, WNT/β‐catenin

## Abstract

Overexpression of TRIP13, a member of the AAA‐ATPase family, is linked with various cancers, but its role in metastasis is unknown in colorectal cancer (CRC). In the current study, we investigated the role TRIP13 in experimental metastasis and its involvement in regulation of WNT/β‐catenin and EGFR signaling pathways. Evaluation of formalin‐fixed paraffin‐embedded (FFPE) and frozen tissues of adenomas and CRCs, along with their corresponding normal samples, showed that TRIP13 was gradually increased in its phenotypic expression from adenoma to carcinoma and that its overexpression in CRCs was independent of patient's gender, age, race/ethnicity, pathologic stage, and p53 and microsatellite instability (MSI) status. Moreover, liver metastases of CRCs showed TRIP13 overexpression as compared to matched adjacent liver tissues, indicating the biological relevance of TRIP13 in CRC progression and metastasis. TRIP13 knockdown impeded colony formation, invasion, motility, and spheroid‐forming capacity of CRC cells irrespective of their p53 and MSI status. Furthermore, xenograft studies demonstrated high expression of TRIP13 contributed to tumor growth and metastasis. Depletion of TRIP13 in CRC cells decreased metastasis and it was independent of the p53 and MSI status. Furthermore, TRIP13 interacted with a tyrosine kinase, FGFR4; this interaction could be essential for activation of the EGFR‐AKT pathway. In addition, we demonstrated the involvement of TRIP13 in the Wnt signaling pathway and in the epithelial–mesenchymal transition. Cell‐based assays revealed that miR‐192 and PNPT1 regulate TRIP13 expression in CRC. Additionally, RNA sequencing of CRC cells with TRIP13 knockdown identified COL6A3, TREM2, SHC3, and KLK7 as downstream targets that may have functional relevance in TRIP13‐mediated tumor growth and metastasis. In summary, our results demonstrated that TRIP13 promotes tumor growth and metastasis regardless of p53 and MSI status, and indicated that it is a target for therapy of CRC.

## Graphical abstract

This study demonstrates overexpression of TRIP13 in colorectal cancers is independent of patient's gender, age, race/ethnicity, pathologic stage (primary and liver metastatic lesions), and p53 and microsatellite instability status. Furthermore, it establishes role of TRIP13 in colorectal cancer metastasis and identifies COL6A3, TREM2, SHC3, and KLK7 as its downstream targets. Additionally, TRIP13 activates EGFR‐AKT pathway *via* its interaction with FGFR4.

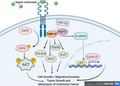

**Abbreviations**
CIN: chromosomal instability
CRC: colorectal cancer
EMT: epithelial–mesenchymal transition
FFPE: formalin‐fixed, paraffin‐embedded
LEF: lymphoid enhancer factor
MS: microsatellite
MSI: microsatellite instable
MSS: microsatellite stable
NSG: NOD/SCID/IL2γ receptor‐null
NT: nontargeting
SAC: spindle assembly checkpoint
TCF: T‐cell factor
TRIP13: thyroid hormone receptor interactor 13
UAB: University of Alabama at Birmingham

## 1 Introduction

Colorectal cancer (CRC) is the third leading cause of cancer deaths in the United States [1]. Chemotherapy remains the standard treatment for advanced CRC [2]. Despite substantial advances in development of adjuvant therapies over the past decade, overall survival has not improved due to the high recurrence rates, intratumoral cellular heterogeneity, and distant metastasis [3]. Therefore, there is an urgent need to identify molecular targets for personalized therapies with efficacy against CRC growth and metastasis.

Accumulation of genetic and epigenetic changes transforms epithelial colon cells into adenocarcinomas [4]. A striking feature of CRCs is chromosomal instability (CIN), which is present in 80–90% of CRCs and leads to microsatellite (MS)‐stable (MSS) tumors [5, 6]. Genetic defects in DNA repair systems predispose cells to cancer susceptibility and tumor development [7]. Additionally, *APC* mutations, oncogenic β‐catenin in the WNT signaling pathway [8], mutations of *TP53,* activation of the RAS/MAPK pathways, and mutations of *RAS* family members contribute to CRC progression [9, 10]. Heterogeneous genetic alterations in *RAS* family members, which are downstream of *EGFR*, are present in about 50% of CRCs [11]. EGFR stimulation results in the activation of β‐catenin/T‐cell factor (TCF)/lymphoid enhancer factor (LEF)‐dependent transcription of genes such as cyclin D1, C‐myc, and survivin [12]. Activation of canonical WNT/β‐catenin signaling is associated with the epithelial–mesenchymal transition (EMT) [13]. During the EMT process, there is an increase in nuclear β‐catenin and TCF [14].

Thyroid hormone receptor interactor 13 (*TRIP13*) is a cancer predisposition gene encoding highly conserved AAA‐ATPase family members, and TRIP13 mutations confer CIN and premature chromosome segregation dysfunctions, leading to aneuploidy [15, 16]. The AAA‐ATPase family of enzymes is involved in diverse biological processes, including DNA replication and protein folding [17]. TRIP13 is a component of the spindle assembly checkpoint (SAC) pathway and, in mitosis, remodels the SAC effector protein, MAD2, to an inactive form [15]. Like poly (ADP‐ribose) polymerase (PARP), TRIP13 mediates double‐strand break repair by alternative error‐prone, nonhomologous end‐joining [18, 19]. Furthermore, TRIP13 fuels repair of chemoresistance of cancer cells and is involved in cellular transformation, leading to progression of head and neck cancers [19]. Overexpression of TRIP13 is associated with a poor prognosis for breast cancer [20], head and neck cancer [19], multiple myeloma [21], glioblastoma [22], and CRC [23]. TRIP13 is part of a 19‐gene signature, identified by Affymetrix array analyses, that predicts survival of patients with CRC [24]. In CRCs, TRIP13 interacts with YWHAZ, a member of the 14‐3‐3 family of proteins, and mediates the G2‐M transition and the EMT [25]. Additionally, TRIP13 is elevated in CRCs, compared to normal colonic tissues, and it facilitates tumor growth and progression [23, 25].

In CRCs, however, regulation of TRIP13 expression, its downstream targets, and its involvement with WNT/β‐catenin and EGFR activation and metastasis, is not known. It is also unknown if its expression is dependent on patient's race/ethnicity, age, and gender, or on the p53 and MS status of tumors. Moreover, it is unclear if the oncogenic properties of CRC cells after TRIP13 knockdown are dependent on MS instability (MSI) or on their p53 status. Thus, the present work focused on elucidating these aspects of CRCs.

## 2 Methods

### 2.1 Colorectal tissue specimens

The Anatomic Pathology Division of the University of Alabama at Birmingham (UAB) provided three cohorts of samples. Cohort 1: Formalin‐fixed, paraffin‐embedded (FFPE) archival tissue blocks representative of benign/normal tissues, and adenomas and adenocarcinomas of the colorectum for immunohistochemical analysis (*n* = 74; Table 1). Samples of this cohort were previously evaluated for MS and p53 mutational status [26, 27]. Cohort 2: Frozen specimens with corresponding normal tissues (*n* = 95) for quantitative PCR (qPCR) and immunoblot analysis as described previously [28]. Cohort 3: Frozen liver metastases of CRCs and matched normal liver specimens (*n* = 9) were used to investigate the expression of TRIP13. All samples were collected and utilized in the experiments after obtaining approval from the UAB Institutional Review Board (IRB number: 060911009). Since utilized samples were the diagnostic remnant tissues, the IRB has waived for Health Insurance Portability & Accountability Act (HIPAA) and individual consenting. The study methodologies conformed to the standards set by the Declaration of Helsinki.

**Table 1:** Clinicopathologic characteristics of CRCs in context with TRIP13 expression.

	IHC (*n* = 74)			qPCR (*n* = 95)		
	High	Low	*P*	High	Low	*P*
	(*N* = 62)	(*N* = 12)		(*N* = 86)	(*N* = 9)	
Age	68.2 ± 11.2	57.3 ± 12.0	0.003	65.6 ± 13.6	67.4 ± 12.3	0.725
Gender						
Female	20 (34.5%)	7 (58.3%)	0.223	43 (50.0%)	3 (37.5%)	0.714*
Male	38 (65.5%)	5 (41.7%)		43 (50.0%)	5 (62.5%)	
Race						
Black	18 (31.0%)	5 (41.7%)	0.707	30 (36.6%)	2 (25.0%)	0.707*
White	40 (69.0%)	7 (58.3%)		52 (63.4%)	6 (75.0%)	
Stage						
I	7 (12.1%)	1 (8.3%)	0.54*	12 (14.0%)	2 (25.0%)	0.409*
II	33 (56.9%)	6 (50.0%)		27 (31.4%)	4 (50.0%)	
III	14 (24.1%)	5 (41.7%)		36 (41.9%)	2 (25.0%)	
IV	4 (6.9%)	0 (0.0%)		11 (12.8%)	0 (0.0%)	
p53 mutation (any)						
N	24 (46.2%)	9 (75.0%)	0.138			
P	28 (53.8%)	3 (25.0%)				
MSI						
MSI‐H	8 (22.2%)	4 (36.4%)	0.585			
MSS	28 (77.8%)	7 (63.6%)				
Tumor grade						
G1	48 (82.8%)	9 (81.8%)	0.962*			
G2	6 (10.3%)	1 (9.1%)				
Mucinous	4 (6.9%)	1 (9.1%)				
Death from cancer						
Alive/other	37 (63.8%)	6 (50.0%)	0.57			
Dead	21 (36.2%)	6 (50.0%)				
All cause death						
Alive	8 (13.8%)	3 (25.0%)	0.592			
Dead	50 (86.2%)	9 (75.0%)				

* Indicates Fisher exact test. IHC and qPCR are performed on two independent cohorts (IHC on retrospective FFPE cohort and qPCR on RNA from frozen tissues of prospective cohort).

### 2.2 Cell lines

McCoy's media (Corning™ Cellgro™; Fisher Scientific Co., Pittsburgh, PA, USA) supplemented with 10% FBS (Invitrogen, ThermoFisher Scientific, Carlsbad, CA, USA) and penicillin–streptomycin was used to grow the following CRC cell lines: HCT116^p53‐wt,MSI^ (RRID:CVCL_0291) and HCT116^p53‐null,MSI^ (RRID:CVCL_HD97) exhibiting MSI‐H; SW480^p53‐mut,MSS^ (RRID:CVCL_0546) and HT29^p53‐mut,MSS^ (RRID:CVCL_0320) cells having MSS. The SW480^p53‐mut^ cell line exhibits double mutations in *p53* at codons 273 and 309: HT29^p53‐mut^ cells exhibit a p53 mutation at codon 273. Cell line identification and authentication were performed by short tandem repeat DNA profiling at the UAB Heflin Center for Genomic Sciences. Cells were also regularly screened for mycoplasma. To generate lentiviruses, the UAB Neuroscience NINDS Vector Core (P30 NS047466) used a pGreenPuro^TM^ shRNA expression lentivector having DNA sequences against TRIP13 (Systembio, Palo Alto, CA, USA). TRIP13 shRNA sequences are given in Table S1.

### 2.3 RNA interference

Lentiviruses expressing TRIP13 shRNA or nontargeting (NT) shRNA were used to transfect CRC cells with 2 μg·mL^−1^ polybrene; stable cell lines were generated by selection with 1 μg·mL^−1^ puromycin (Life Technologies, ThermoFisher Scientific, Carlsbad, CA, USA) as described previously [29, 30]. Selected cells were used for immunoblot analyses and cell‐based functional assays.

### 2.4 Quantitative real‐time PCR (qPCR)

For total RNA isolation from CRC tissues and cell lines, RNeasy mini kits (Zymo Research, Irvine, CA, USA) were used as described previously [31]. RNA was converted into cDNA using Superscript III Reverse Transcriptase (Invitrogen), followed by qPCR using SYBR green and primers synthesized by Integrated DNA Technologies (Coralville, IA, USA) to determine the mRNA expression of *TRIP13*, *COL6A3*, *TREM2*, *SHC3*, and *KLK7* in CRC cells and tissues. β‐Actin was used as a normalizing control. A list of primers used in this study is given in Table S2.

### 2.5 Immunoblot analyses

As described earlier [28, 29], immunoblot analyses were accomplished to evaluate TRIP13 protein expression in CRC cells. SDS/PAGE involved use of NuPAGE™ 4–12% Bis‐Tris Midi Protein Gels, 20‐well (Invitrogen, ThermoFisher Scientific). Protein samples were separated followed by transfer onto Immobilon‐P poly(vinylidene difluoride) membranes (EMD Millipore, Billerica, MA, USA). Blocking buffer [Tris‐buffered saline, 0.1% Tween (TBS‐T), 5% nonfat dry milk] was used to block nonspecific binding sites followed by overnight incubation with primary antibodies at 4 °C. Horseradish peroxidase‐conjugated secondary antibody was used to probe blots for 1 h, and Luminata^TM^ Crescendo chemiluminescence western blotting substrate was used to visualize signals according to the manufacturer's protocol (EMD Millipore). A list of primary antibodies, along with their catalog and RRID numbers, is provided in Table S3.

### 2.6 Immunoprecipitation analysis

Colorectal cancer cells were collected in Pierce IP lysis buffer (Cat# 87787; Invitrogen, ThermoFisher Scientific) containing HALT protease and phosphatase inhibitors cocktail (Cat# 78440; Invitrogen, ThermoFisher Scientific). After centrifugation, the cell lysates (1 mg·mL^−1^) were collected. The protein A/G magnetic beads (ThermoFisher Scientific, Waltham, MA, USA) were incubated with cells at 4 °C for 2 h. After washing three times with buffer, 5 µg of anti‐TRIP13 antibody or anti‐EGFR or rabbit control IgG (Table S3) was added to the beads having cell lysates, followed by the incubation of antibody‐bound magnetic beads and cell lysates at 4 °C for overnight. The pellet with magnetic bead was washed three times with buffer and then resuspended in IP lysis and loading buffer, followed by incubation at 100 °C for 10 min. Western blot using anti‐TRIP13 antibody was performed to find the TRIP13 protein as described above. Samples were analyzed for mass spectrometry analysis.

### 2.7 Immunohistochemical analysis

To evaluate TRIP13 protein expression, immunohistochemical (IHC) analysis was performed on CRC tissue specimens as described earlier [32]. TRIP13 antibody (Cat# 19602‐1‐AP, RRID: AB_10642702, Proteintech Group, Rosemont, IL, USA; Table S3) was used to probe tumor sections after deparaffinization, rehydration, antigen retrieval by EDTA buffer, and blocking with horse serum. Further, ImmPRESS HRP anti‐mouse IgG (Cat# MP‐7402, RRID: AB_2336528; Vector laboratories, Burlingame, CA, USA), as a secondary antibody, was used to probe tumor sections. Diaminobenzidine (Cat# SK‐4100, RRID: AB_2336382; Vector laboratories) was used to assess immunoreactivity. For nuclear staining, vector Hematoxylin QS (Cat#H‐3404; Vector labs) was used.

### 2.8 Cellular proliferation assay

To assess cellular proliferation, stable TRIP13 knockdown and control NT shRNA‐transfected CRC cells (5 × 10^3^) were seeded into 24‐well plates in triplicate. Cell counting was accomplished at 0, 2, 4, and 6 days with a Z2 Coulter particle counter (Beckman Coulter, Brea, CA, USA).

### 2.9 Colony formation assay

Colony formation assays were performed using stable TRIP13 knockdown and NT shRNA‐transfected CRC cells, as described [33]. Cells (1 × 10^3^) were seeded into 6‐well plates in triplicates; after 10 days, cells were fixed with 5% glutaraldehyde and stained with crystal violet (Sigma‐Aldrich, St. Louis, MO, USA). Cells were washed with PBS, and images of colonies were acquired by Amersham Imager 600RGB (GE Healthcare Life Sciences, Pittsburgh, PA, USA).

### 2.10 Invasion assay

Cell invasion assays were performed with Corning BioCoat^TM^ Matrigel matrix (Corning, NY, USA) as described previously [34, 35]. Stable TRIP13 knockdown or NT shRNA‐transfected CRC cells (5 × 10^4^) in triplicate were layered onto 8‐μm pore inserts in 24‐well plates in 500 μL of serum‐free medium. Chemoattractant medium (750 μL, supplemented with 10% FBS) was added in the lower chambers. After 48 h, noninvading cells and the Matrigel matrix were removed with a cotton swab. Invaded cells were fixed with 5% glutaraldehyde and stained with crystal violet. A phase‐contrast microscope was used to take images.

### 2.11 Wound‐healing assay

Stable TRIP13 knockdown and NT shRNA‐control CRC cells (1 × 10^6^) were seeded onto 35‐mm Petri dishes as described [36]. An artificial wound was created on the confluent cell monolayers using tips of 200‐μL pipets. At 0 and 24 h, images were taken with an inverted phase‐contrast microscope with a 4X objective.

### 2.12 Spheroid 3D model

Cultrex® 3D spheroid BME cell invasion kits (Cat# 3500‐096‐K; Trevigen, Gaithersburg, MD, USA) were used to establish CRC spheroids as described earlier [29]. Cells (1 × 10^4^), in triplicates, were seeded onto 96‐well plates with 5 µL of Spheroid Formation ECM according to the manufacturer's instructions. The cells were centrifuged at 200 ***g*** for 3 min to move spheroids toward the middle of the wells; the cells were then incubated in a 37 °C, 5% CO_2_ incubator for 72 h. At this time, invasion matrix (50 μL) was added to the wells. The preparations were centrifuged at 200 ***g*** for 3 min at 4 °C, and warm media containing FBS was added. After 4 days of incubation, images were taken with 4X objective.

### 2.13 miRNA luciferase binding assay

As described [29, 37], 3′‐UTR luciferase assays were performed. pMIR‐REPORT miRNA Expression Reporter Vector (Life Technologies) was used to clone wild‐type or mutant TRIP13 3′‐UTRs. Pre‐miR‐192 or NT miR (#AM17110) was transfected using Lipofectamine 2000 into HCT116^p53‐wt,MSI‐H^ cells for 4 h, followed by transfection with wild‐type or mutant 3′‐UTR‐luc, as well as with a pRL‐TK vector as an internal control for luciferase activity. The cells were lysed at 72 hr post‐transfection, and the dual luciferase assay system (Promega, Madison, WI, USA) was used to conduct renilla luciferase assays as per the manufacturer's instructions. Each experiment was performed in triplicate.

### 2.14 Tumor xenograft and metastasis model

As described earlier [31, 38], for tumor xenograft experiments, NOD/SCID/IL2γ receptor‐null (NSG) mice (*n* = 6 for each group) were injected subcutaneously into the right dorsal flanks with of HCT116^p53‐wt^ cells (1 × 10^6^ cells in 50 µL of incomplete media without FBS, and 50 µL of Matrigel) with or without transfection with TRIP13 shRNA. Animal experiments were approved by the Institutional Animal Care and Use Committee of UAB. After inoculation of the cells, tumor growth was measured with Vernier calipers and recorded on a weekly basis. Tumor volume was calculated with formula: 0.5 × tumor length × tumor width^2^. Four weeks after inoculation of cells, tumor tissues were excised, imaged, and weighed. Tissues were frozen for western blot analysis.

For experimental metastasis studies, highly metastatic, luciferase tagged HCT116^p53‐wt,MSI‐H^ and HT29^p53‐mut,MSS^ cells transfected with NT or TRIP13 shRNA (0.5 × 10^6^ cells in 100 µL of incomplete media without FBS) were injected in the lateral tail veins of NSG mice as described previously [36, 39]. For imaging of mice, the *In Vivo* IVIS Lumina Series III spectrum imaging system (Perkin Elmer, Waltham, MA, USA) was used to measure luciferase activity noninvasively. d‐Luciferin (100 μL per mouse of 10 mg·mL^−1^ dissolved in PBS) was injected intraperitoneally into mice, 10 min before luminescence imaging. All mice were placed in the imaging instrument, scanned, and exposed at the same time. Mice were euthanized 4 weeks later, and their organs were removed for assessment of metastases. Immediately before sacrifice, mice were injected intraperitoneally with luciferin to facilitate *ex vivo* assessments of bioluminescence. Organs with metastases were retrieved and embedded in paraffin.

### 2.15 Bone marrow cultures

Bone marrow containing cancer cells was flushed from the femurs and tibias of mice with a 1‐mL syringe. Bone marrow cells from mice injected with HT29^p53‐mut,MSS^ cells transfected with NT or TRIP13 shRNA were cultured as described previously [36] for 21 days. At 8 and 21 days, an inverted microscope was used to take bright‐field and green fluorescent protein (GFP) images of the cells.

### 2.16 Immunofluorescence costaining of bone specimens from mice

Bone specimens from mice were fixed, decalcified, and processed for paraffin embedding. An IHC protocol was followed as described previously [36]. Briefly, bone sections were deparaffinized, rehydrated, and antigen retrieved. Sections were blocked and incubated with cytokeratin 8 + 18 and alkaline phosphatase (Table S3) for 1 h, followed by incubation with secondary antibodies (anti‐mouse IgG Alexa Fluor 647 and anti‐rabbit IgG Alexa Fluor 555) for 1 h. Prolong® Gold Antifade reagent with 4′,6‐diamidino‐2‐phenylindole (DAPI, Cat#P36931, Life technologies, Eugene, OR, USA) was used for nuclear staining and mounting of specimens. Confocal images using a 60X lens Nikon A1, High Speed Laser Confocal Spectral Imaging microscope (Nikon Instruments Inc., Melville, NY, USA) were acquired at the UAB High‐Resolution Imaging Facility. All image acquisitions were performed by keeping constant detector gains.

### 2.17 Statistical analysis

We analyzed two sets of samples: (a) 74 paired normal‐tumor FFPE samples by IHC, and (b) 95 paired normal‐tumor frozen samples by qPCR. The hypothesis was that IHC/qPCR values would differ between tumor and normal paired samples. To compare mean measures between groups, we used a Wilcoxon matched‐pairs signed rank test, and we performed a subanalysis for all demographic variables with the same test statistic. For IHC samples, an additional comparison was made between CRC and normal samples using an unmatched Wilcoxon test. Where indicated, Fisher's exact tests and *t*‐tests were used for categorical analyses. MS (MSI‐H vs. MSS) and p53 (wild‐type vs. mutant) CRCs (FFPE cohort) were evaluated separately for correlation with TRIP13 IHC scores for tumor and normal tissues using Spearman rank correlations. A *P*‐value of < 0.05 was considered statistically significant. For assays involving cells in culture, data were expressed as means ± standard deviation for triplicates.

## 3 Results

### 3.1 Overexpression of TRIP13 in CRCs is independent of cancer stage, patient's race/ethnicity, gender, and age

**Fig 1:**
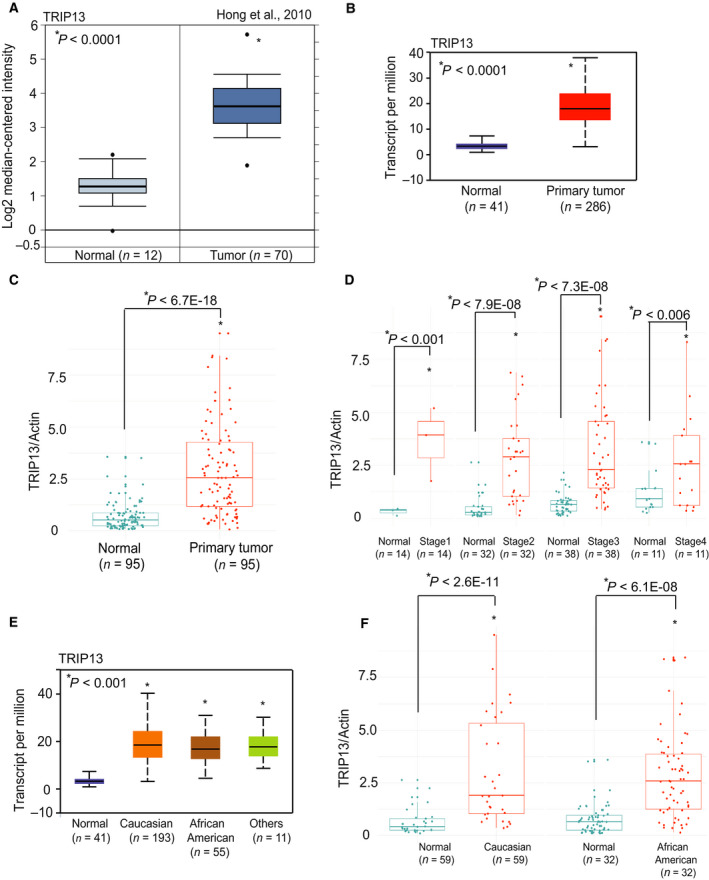
Overexpression of the *TRIP13* gene in CRCs is independent of tumor stage, and patient's race, gender, and age. (A) TRIP13 expression in colon adenocarcinoma tissues using Oncomine gene expression profiling. (B) UALCAN data showing TRIP13 expression in colorectal adenocarcinomas (*n* = 286) compared to normal colon (*n* = 41). (C) TRIP13 mRNA expression in 95 CRC tissues and corresponding adjacent noncancerous tissues by employing qPCR analysis. (D) Stage‐wise expression of TRIP13 in paired samples of colorectal adenocarcinoma and normal colon [Stage 1 (*n* = 14), Stage 2 (*n* = 32), Stage 3 (*n* = 38) and Stage 4 (*n* = 11)]. (E) Boxplot analysis of data acquired from UALCAN showed TRIP13 expression in Caucasians (*n* = 193) and African Americans (*n* = 55). (F) Quantitative PCR analysis of TRIP13 mRNA in matched tissues of CRC and normal colon with respect to race of patients [Caucasian (*n* = 59) vs African American (*n* = 32)]; *P < 0.01.

Publicly available Oncomine 3 [Oncomine™ Platform (Life Technologies, Ann Arbor, MI, USA) data showed high expression of *TRIP13* in CRCs (Fig. 1A)] [40]. Gene expression profiling of CRCs using UALCAN (http://ualcan.path.uab.edu/) [41] showed elevated *TRIP13* expression (Fig. 1B). Further, in‐house validation, accomplished by performing qPCR analysis of frozen samples of paired tumor and normal tissues (*n* = 95) found *TRIP13* overexpression in CRCs as compared to matched normal colon (Fig. 1C). TCGA data acquired from UALCAN showed that *TRIP13* overexpression was independent of pathologic stage of the tumor (Supplementary Fig. 1A). By qPCR analysis, we demonstrated that *TRIP13* mRNA expression was elevated in CRCs of different stages [Stage 1 (*n* = 14), Stage 2 (*n* = 32), Stage 3 (*n* = 38), and Stage 4 (*n* = 11); Fig. 1D]. UALCAN showed that TRIP13 overexpression was independent of patient's race (Fig. 1E), gender (Supplementary Fig. 1B), age (Supplementary Fig. 1C), and weight (Supplementary Fig. 1D). qPCR analysis confirmed our *in silico* finding of TRIP13 overexpression regardless of patient's race [Caucasian (*n* = 59) vs African American (*n* = 32)] (Fig. 1F); gender [male (*n* = 48) vs female (*n* = 47); Supplementary Fig. 1E]; and age [21–40 years (*n* = 3), 41–60 years (*n* = 26), 61–80 years (*n* = 47) and 81–100 years (*n* = 19); Supplementary Fig. 1F]. Thus, *TRIP13* overexpression in CRCs was independent of the pathologic stage and the race, age, and gender of patients.

### 3.2 Protein expression of TRIP13 in colorectal adenocarcinomas is regardless of p53 and microsatellite status

**Fig 2:**
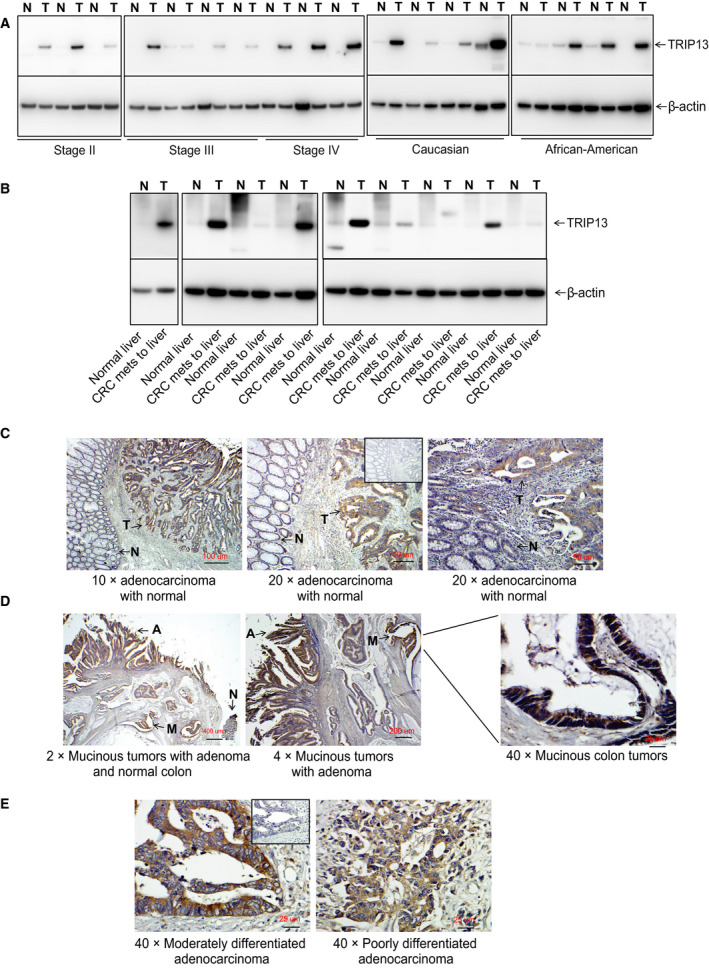
TRIP13 protein expression in CRCs. Immunoblot analysis for TRIP13 protein expression in CRC tissues relative to normal colon in (A) different tumor stages and race of patients. β‐Actin was used as a loading control. (B) TRIP13 protein expression in lysates of liver metastases of CRCs and adjacent liver controls. Representative images showing IHC staining with TRIP13‐specific antibody in tissue specimens containing (C) tumor (denoted by T) and adjacent normal (denoted by N) tissues and (D) mucinous adenocarcinoma (denoted by M), adenoma (denoted by A), and normal colon (denoted by N). (E) TRIP13 staining in CRC specimens based on moderate and poor differentiation. Scale bar; 2×—400 µm, 4×—200 µm, 10×—100 µm, 20×—50 µm, 40×—25 µm.

There was higher expression of the TRIP13 protein in frozen CRCs relative to their matched normal/benign tissues, and TRIP13 overexpression was independent of tumor stage and patient race (Fig. 2A). Moreover, TRIP13 overexpression was evident in liver metastases of CRCs as compared to adjacent normal liver tissues (Fig. 2B). Furthermore, IHC analysis was performed using a colon tissue microarray containing normal, adenoma, and tumor tissue. Patient samples (169, IHC = 74, qPCR = 95) were used in the analysis (Table 1). There was higher expression of the TRIP13 protein in tumor tissues relative to their matched normal tissues (Fig. 2C; Wilcoxon *P*‐values 6.10e−10; Supplementary Fig. 2A). In mucinous adenocarcinomas, there was high expression of TRIP13 (Fig. 2D), as was the case for all tumor grades (Fig. 2E). Consistent with data on the mRNA expression of TRIP13, its immunophenotypic expression was independent of tumor stage (Supplementary Fig. 2B) and patient race (Supplementary Fig. 2C), gender (Supplementary Fig. 2D), and age (Supplementary Fig. 2E). A gradual increased expression was noted from adenoma (mid), and tumor (high) tissues (Kruskal–Wallis *P*‐value 1.10e−8). Differences between tumor and normal samples were measured across the tumor and demographic variables and p53 (wt vs. mutation) and MS status (MSI‐H and MSS). TRIP13 IHC scoring was higher for tumors, and no modifying effect was evident within the categories of each variable (Table 1). These results showed that TRIP13 overexpression in CRCs is independent of p53 and MS status.

### 3.3 TRIP13 loss impairs CRC malignant phenotypes irrespective of their p53 and microsatellite status

**Fig 3:**
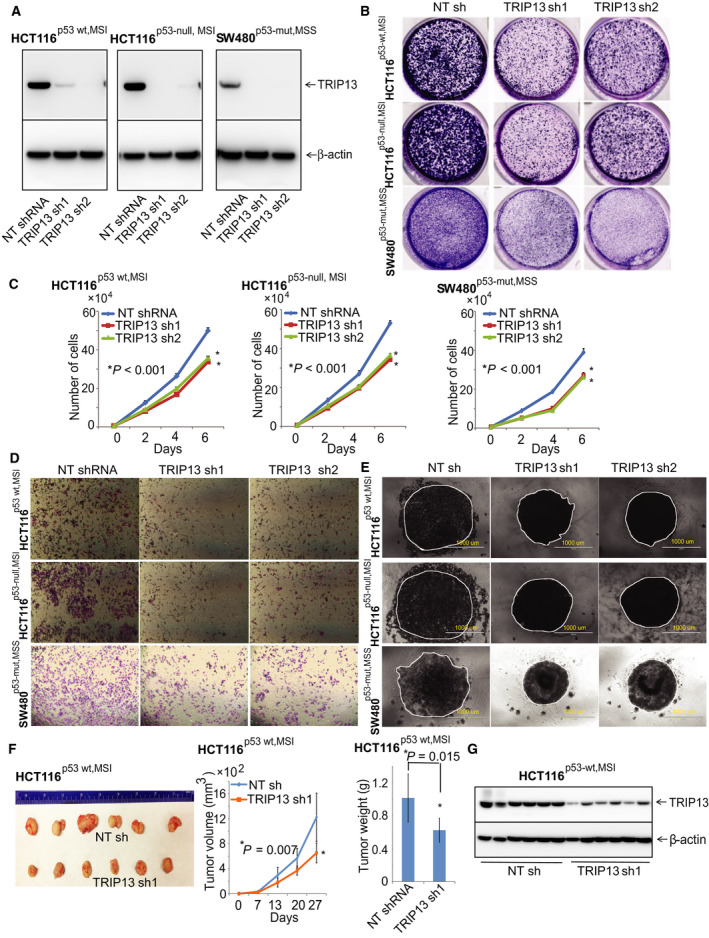
TRIP13 promotes malignant phenotypes and growth of CRC cells. (A) Immunoblot analysis showed TRIP13 protein ablation in CRC cell lysates compared to results for NT shRNA. β‐Actin was used as a loading control. (B) TRIP13 knockdown in CRC cell lines impeded colony formation and cell proliferation (C). (D) Transwell Matrigel invasion assay showing results for TRIP13‐ablated and control NT shRNA CRC cells. (E) CRC spheroids formed after TRIP13 knockdown as compared with cells transfected with NT shRNA; scale bar, 1000 µm. For all these experiments, values are mean ± SD (error bars) of triplicate samples from a representative experiment. (F) NSG mice were inoculated with HCT116^p53‐wt,MSI^ cells transfected with control NT or TRIP13 shRNA (*n* = 6). Tumor sizes were measured each week. At the end of the experiment, tumors were retrieved after euthanization of mice. Images of excised xenograft tumors, tumor volumes, and weight are shown in a representative figure. Asterisk (*) shows statistically significant data (*P < 0.05). (G) Tumor lysates from xenografts were subjected to immunoblot analysis and probed with TRIP13. β‐Actin was used as an internal loading control.

We investigated the functional role of TRIP13 in CRC cells with different p53 and MSI status. TRIP13 knockdown was accomplished using a lentiviral delivery system for HCT116^p53‐wt,MSI^, HCT116^p53‐null,MSI^, and SW480^p53‐mut,MSS^ cells (Fig. 3A). Colony formation and cell proliferation assays revealed that, for cells lacking TRIP13, their capacity to form colonies and their proliferation rate were low (Figs. 3B,C). Further, we assessed lack of TRIP13 on migration and invasion of CRC cells. Transwell invasion assays were used to investigate TRIP13 ablation on the invasive properties of CRC cells. HCT116^p53‐wt,MSI^, HCT116^p53‐null,MSI^, and SW480^p53‐mut,MSS^ cells had low capacity for invasion when they were transfected with TRIP13 shRNA1 or TRIP13 shRNA2 compared to cells transfected with NT shRNA (Fig. 3D). We next performed scratch‐wound assays in which time‐lapse microscopy assessed the capacity of cancer cells to migrate and fill an artificial wound. A wound was created in confluent monolayers of TRIP13‐depleted CRC cells, and images were acquired at 0 and 24 h. These assays revealed that, after TRIP13 knockdown, the capacity of CRC cells to migrate and fill in a wound was impaired (Supplementary Fig. 3). These results were consistent with the observation that knocking down TRIP13 inhibited the invasive capacity of CRC cells. As 3D spheroid cultures imitate features of tumors, we performed spheroid assays to understand the biological role of TRIP13 in CRC spheroid formation. Loss of TRIP13 reduced the growth of CRC spheroids (Fig. 3E). These data confirmed that CRC growth inhibition after TRIP13 knockdown is independent of p53 and MSI status.

To investigate the role of TRIP13 on CRC growth in mice, we established a xenograft model involving implantation of stably transfected HCT116^p53‐wt,MSI‐H^ cells with or without reduced TRIP13 expression. A representative photograph shows tumors from HCT116^p53‐wt,MSI^ tumors transfected with NT shRNA or TRIP13 shRNA (Fig. 3F). The results indicated that xenograft tumors induced by cells transfected with shTRIP13 were smaller than tumors induced by cells transfected with control NT shRNA (Fig. 3F). In addition, there were lower weights of tumors formed by cells transfected with a TRIP13 shRNA as compared to those formed by cells transfected with NT shRNA (Fig. 3F). In concurrence with data from *in vitro* analysis of TRIP13 knockdown, xenograft lysates subjected to immunoblot analysis also showed low expression of TRIP13 in TRIP13 knockdown cells (Fig. 3G). These data show that a TRIP13 deficiency reduces the malignant phenotypes of CRC growth.

### 3.4 TRIP13 knockdown reduces CRC metastasis regardless of the p53 and microsatellite status of cells

**Fig 4:**
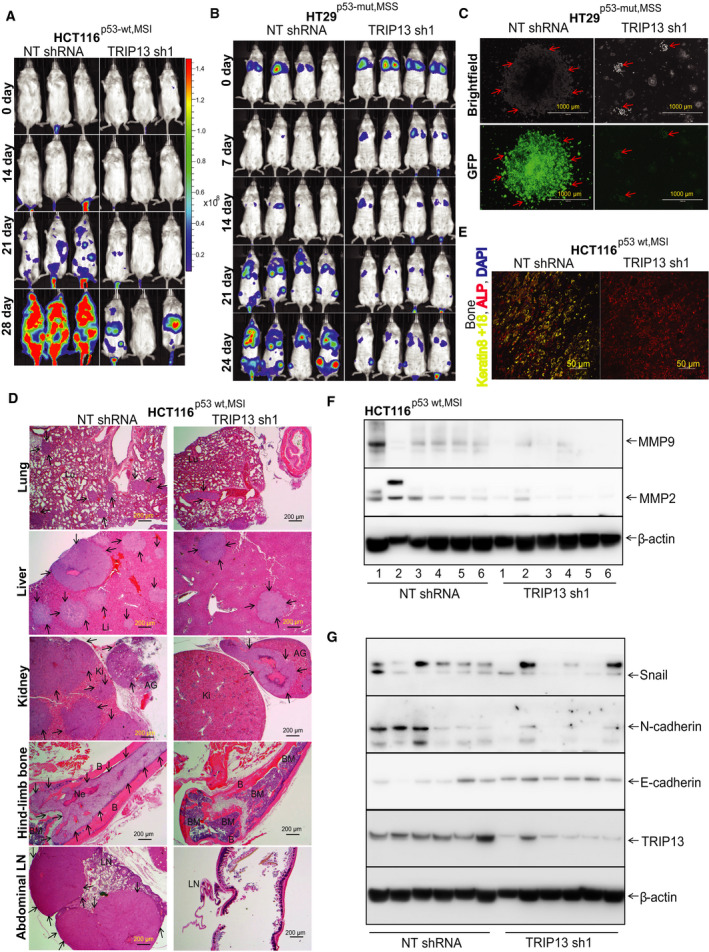
TRIP13 depletion association with low metastasis of CRCs is independent of p53 and MS status. (A) Bioluminescence images of representative animals of each group at days 0, 14, 21, and 28 after injection of HCT116^p53‐wt,MSI^ cells and (B) 0, 7, 14, 21, and 24 days for HT29^p53‐mut,MSS^ cells. (C) Bone marrow of mice injected with HT29^p53‐mut,MSS^ cells was flushed and cultured in media. Representative phase‐contrast and GFP images after 21 days showing bone marrow or cancer cells procured from bone marrow of mice injected with HT29^p53‐mut,MSS^ cells transfected with NT shRNA or with TRIP13 shRNA; 1000 µm. (D) H&E images of lung, liver, kidney, hind‐limb bone, and abdominal lymph nodes from HCT116^p53‐wt,MSI^ cells transfected with control NT or TRIP13 shRNA. Arrow shows metastatic lesions; Lu—lung; Li—liver; Ki—kidney; AG—adrenal gland; B—bone; BM—bone marrow; Ne—necrosis; LN—lymph nodes. 4×—scale bar, 200 µm. (E) Immunofluorescence costaining of keratin 8 + 18 (yellow) and ALP (red) on HCT116^p53‐wt,MSI^ bone lesions. DAPI was used for nuclear staining. (F) Western blots of HCT116^p53‐wt,MSI^ xenograft lysates exhibiting TRIP13 knockdown showing the expressions of MMP2 and MMP9. (G) Xenograft lysates exhibiting NT shRNA or TRIP13 shRNA were probed with E‐cadherin, N‐cadherin, Snail, and TRIP13 antibodies. For the experiment, β‐actin was used as a loading control.

To determine whether TRIP13 knockdown affects metastasis, luciferase‐labeled HCT116^p53‐wt,MSI^ and HT29^p53‐mut,MSS^ cells transfected with TRIP13 shRNA or with NT shRNA were injected into the tail veins of NSG mice, as described [36, 39]. To assess TRIP13 knockdown efficiency, luciferase‐labeled HT29^p53‐mut^ cells were transfected with or without TRIP13 shRNA followed by western blot analysis (Supplementary Fig. 4A). The capacity of the HCT116^p53‐wt,MSI^ and HT29^p53‐mut,MSS^ cells to form metastases was examined by noninvasive bioluminescence imaging of mice, which allowed chronological monitoring of tumor growth. Based on luminescence intensity, metastases were more extensive for mice injected with HCT116^p53‐wt,MSI^ cells as compared to HT29^p53‐mut,MSS^ cells transfected with NT shRNA. The results indicated that TRIP13 silencing led to lower luminescence signaling in distant organs of mice injected with HCT116^p53‐wt,MSI‐H^ (Fig. 4A) or HT29^p53‐mut,MSS^ cells (Fig. 4B) as compared to that for control mice injected with cells transfected with NT shRNA. In mice injected with HCT116^p53‐wt,MSI^ cells, necropsies showed inguinal lymph node metastasis and metastatic nodules in the liver, near the armpit, or in the thoracic areas of mice injected with cells transfected with NT shRNA as compared to mice injected with cells transfected with TRIP13 shRNA (Supplementary Fig. 4B). Luciferase signals obtained by *ex vivo* imaging at 4 weeks after injection were evident in lungs, liver, kidney, and intestine, but signals were lower for mice injected with cells transfected with TRIP13 shRNA than for mice injected with cells transfected with NT shRNA (Supplementary Fig. 4C). For this experimental model, liver, lungs, kidney, and bone were common sites of metastasis of CRCs. Peritoneal metastases were also evident; these may have spread through the fluid within the peritoneal cavity (Supplementary Fig. 4C). Lymph node metastasis was confirmed by the presence of luminescence signals in lymph nodes of the inguinal (Supplementary Fig. 4D). For mice injected with HCT116^p53‐wt,MSI^ cells transfected with NT shRNA, there were metastases in the thorax; these were confirmed by luminescence signals (Supplementary Fig. 4D). For mice injected with HT29^p53‐mut,MSS^ cells, there were, after 8 (Supplementary Fig. 4E) and 21 days of culture (Fig. 4C), reduced numbers of cancer cells in cultures from bone marrow of mice injected with TRIP13 knockdown cells as compared to cells transfected with NT shRNA. H&E examination validated decreased colonization of in lungs, liver, kidney, and bone marrow of HCT116^p53‐wt,MSI^ (Fig. 4D) and HT29^p53‐mut,MSS^ cells (Supplementary Fig. 5). H&E staining showed infiltration of cancer cells into adipose tissue and skeletal muscles in the neck area and in inguinal lymph nodes (Supplementary Fig. 6). Further, for HCT116^p53‐wt,MSI^ cells, we performed immunofluorescent assays to show the interaction of bone marrow with these cells and validated the presence of metastatic cancers in bone (Fig. 4E). Bone marrow cells were stained with alkaline phosphatase (a marker of bone marrow cells); cancer cells contained cytokeratin 8 + 18 as described by us recently [36, 39]. The results showed marked bone colonization by cancer cells transfected with NT shRNA as compared to cells transfected with TRIP13 shRNA (Fig. 4E). Lysates of subcutaneous xenografts with TRIP13 knockdown also showed lower levels of MMP2 and MMP9, confirming the association of TRIP13 with MMPs in the invasion and metastasis of CRC cells (Fig. 4F). In sum, these results highlight the involvement of TRIP13 in metastasis of CRCs and demonstrate that the less extensive CRC metastasis after TRIP13 knockdown is independent of p53 and MSI status.

**Fig 5:**
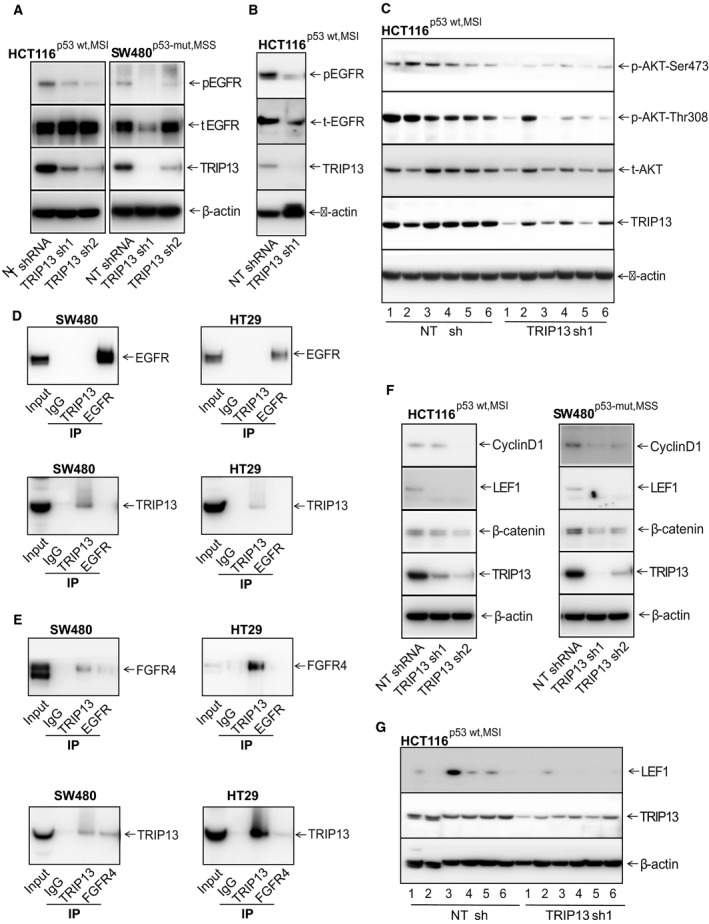
TRIP13 regulates EGFR activation and Wnt signaling. (A) TRIP13‐deficient CRC cell lysates showed decrease in EGFR phosphorylation (EGFR‐Y1068). (B) Western blot analysis to check expression of p‐EGFR‐Y1068, total‐EGFR, and TRIP13 in lysates of bone marrow collected from mice injected with HT29^p53‐mut,MSS^ cells transfected with NT shRNA or TRIP13 shRNA. (C) Western blot analysis to investigate p‐Akt at Ser 473 and Thre‐308 expression in xenograft lysates of HCT116^p53‐wt,MSI^ transfected with NT shRNA or TRIP13 shRNA. Lysates were probed with Akt‐Ser 473, Akt‐Thr308, total‐Akt, TRIP13, and β‐actin. (D) Immunoprecipitation pull downs of control IgG, TRIP13, and EGFR followed by western blot analysis to assess the interaction of TRIP13 with EGFR. (E) Immunoprecipitation of control IgG, TRIP13, EGFR, and FGFR4 followed by western blot analysis of FGFR4 and TRIP13. (F) Western blot of Wnt/β‐catenin pathway proteins expression in control or TRIP13 shRNAs lysates. (G) LEF1 protein expression in HCT116^p53‐wt,MSI^ xenograft lysates exhibiting TRIP13 knockdown.

**Fig 6:**
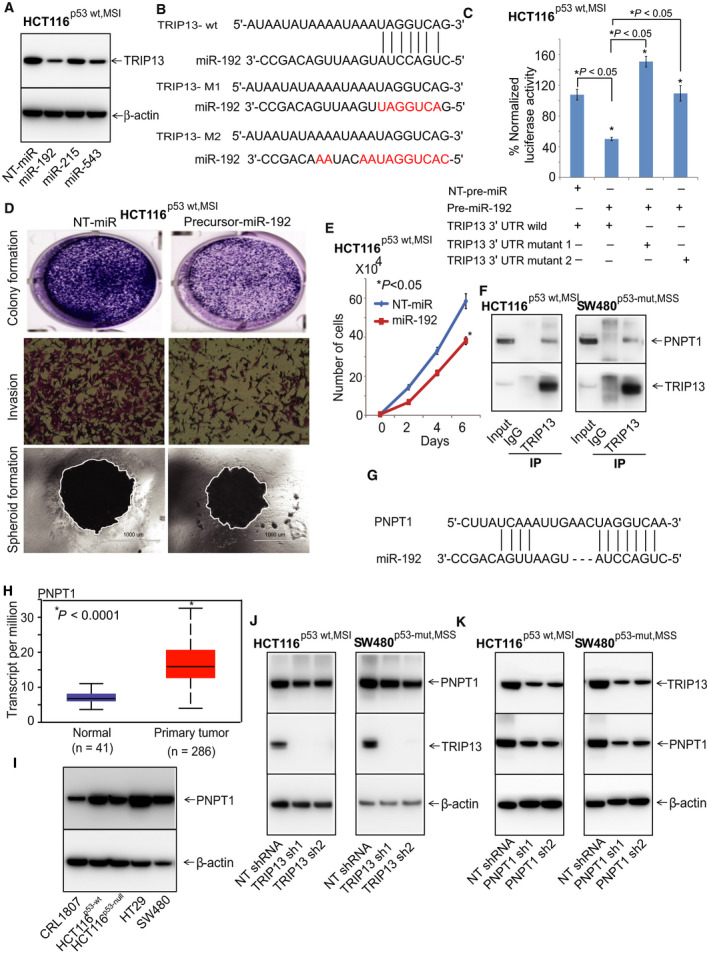
miR‐192 and PNPT1 regulate TRIP13 in CRCs. (A) HCT116^p53‐wt,MSI^ cells were treated with pre‐miR‐192 and checked for TRIP13 protein using immunoblot analysis. (B) Binding site of miR‐192 with TRIP13. (C) Luciferase reporter assay of TRIP13‐3′‐UTR. HCT116^p53‐wt,MSI^ cells transfected with pre‐miR‐192 or NT‐miRNA along with either TRIP13 3′‐UTR wild‐type or mutant. Precursor‐miR‐192‐treated cells were used in assays for (D) colony formation, invasion, spheroid‐forming capacity and (E) cell proliferation; scale bar, 1000 µm. (F) Western blot of TRIP13 immunoprecipitation in CRC cells. PNPT1 was probed with TRIP13‐pull down lysates. Control IgG was used as a negative control while inputs have the raw lysates of CRC cells. (G) Binding sites of miR‐192 with PNPT1. (H) UALCAN analysis to show the RNA expression of PNPT1 in CRC (*n* = 286) and normal colon (*n* = 41) tissues. (I) Western blots to show the expression of PNPT1 in HCT116^p53‐wt,MSI^, HCT116^p53‐null,MSI^, SW480^p53‐mut,MSS^, HT29^p53‐mut,MSS^, and CRL1807 cells. (J) Western blot to show the protein expression of PNPT1 in TRIP13 knockdown CRC lysates, and (K) TRIP13 in PNPT1 knockdown lysates; *P < 0.05.

To confirm the role of TRIP13 in the EMT of CRCs, we performed western blot analysis using lysates of subcutaneous tumor xenografts of HCT116^p53‐wt,MSI^ cells and probed for the epithelial marker, E‐cadherin, and the mesenchymal markers, N‐cadherin, and Snail. The analysis showed that E‐cadherin was higher and that N‐cadherin and Snail were lower in lysates of xenografts of cells with TRIP13 knockdown (Fig. 4G), indicating the role of TRIP13 in the progression of CRC through the EMT pathway.

### 3.5 The TRIP13‐FGFR4 interaction is involved in the EGFR‐AKT signaling pathway in CRCs

Expressions of EGFR and β‐catenin are upregulated in CRCs, and there is crosstalk between EGFR and WNT/β‐catenin signaling pathways that may be involved in regulation of cell proliferation and survival [10]. For CRC cells, TRIP13 knockdown caused low EGFR phosphorylation at the Tyr1068 residue (Fig. 5A). We also confirmed the down‐regulation of p‐EGFR levels in bone marrow samples obtained from mice bearing TRIP13‐knockdown cells (Fig. 5B). Furthermore, the expression of phosphorylated AKT, a downstream molecule of EGFR, was evaluated in lysates of xenografts with TRIP13 knockdown. After TRIP13 knockdown, there was lower expression of Akt phosphorylation at serine 473 and threonine 308 sites as compared to NT shRNA controls (Fig. 5C). This indicates that TRIP13 is involved in the EGFR/Akt signaling pathway. Since a prior study shows TRIP13 interaction with EGFR [42], we performed an immunoprecipitation assay to demonstrate the interaction between TRIP13 and EGFR using CRC cells, SW480^p53‐mut,MSS^ and HT29^p53‐mut,MSS^. The results showed that there was no direct interaction between EGFR and TRIP13 (Fig. 5D). Further, a previous study of colon cancer [43] shows activation of the EGFR signaling pathway through fibroblast growth factor receptor 4 (FGFR4), a tyrosine kinase. Thus, we probed FGFR4 antibody with EGFR or TRIP13 pulldowns and checked for interaction of FGFR4 with TRIP13 and/or EGFR. In lysates of SW480^p53‐mut,MSS^ and HT29^p53‐mut,MSS^ CRC cells, there was an apparent interaction between TRIP13 and FGFR4 (Fig. 5E), indicating the involvement of TRIP13 in activation of the EGFR signaling pathway through FGFR4. Although control cells retained the expression of *WNT* targets, there was, after *TRIP13* knockdown, a reduction in β‐catenin, LEF1, and cyclin D1 (Fig. 5F). Xenograft lysates showed decreased levels of LEF1 in TRIP13 knockdown CRC xenograft tissues (Fig. 5G).

### 3.6 miR‐192 and PNPT1 regulate TRIP13 in CRC cells

Target prediction using Targetscan (http://www.targetscan.org/vert_72/) showed that miR‐192, miR‐215, and miR‐543 interact with the 3′‐untranslated region (3′‐UTR) of TRIP13 mRNA and may regulate *TRIP13* expression. HCT116^p53‐wt,MSI^ cells were transfected with miR‐192 or with another miRNA, miR‐215 or miR‐543, to determine the predicted miRNA that specifically suppresses TRIP13 expression. Western blot analysis showed lower levels of TRIP13 after miR‐192 overexpression (Fig. 6A). Figure 6B shows the binding site of miR‐192 to the 3′‐UTR of TRIP13. Luciferase reporter vectors containing the 3′UTR of *TRIP13* with the miR‐192 target site in either its wild‐type or a mutated form were coexpressed with miR‐192 in HCT116^p53‐wt,MSI^ cells, and the luciferase activity was measured. There were changes in the luciferase assay for the wild‐type form of *TRIP13*, but not for the mutant, providing evidence for a role of miR‐192 in the regulation of *TRIP13* expression (Fig. 6C). Transient overexpression of precursor‐miR‐192 also resulted in reduced colony formation, invasion, formation of spheroids (Fig. 6D), and cell proliferation (Fig. 6E) by HCT116^p53‐wt,MSI^ cells, indicating a tumor suppressor function of miR‐192. Based on these findings, our hypothesis that TRIP13 was targeted by miR‐192 in HCT116^p53‐wt,MSI^ cells was validated.

Immunoprecipitation in combination with mass spectrometry was performed on HCT116^p53‐wt,MSI^ and SW480^p53‐mut,MSS^ lysates to identify the interacting partners of TRIP13. Mass spectrometric analysis predicted PNPT1 as one of the interacting partner of TRIP13 and western blot analysis confirmed that TRIP13 interacts with PNPT1 (Fig. 6F). Additionally, the Targetscan bioinformatics website found that miR‐192 also binds with 3′‐UTR of PNPT1 and has the same binding sites through which it binds to TRIP13, suggesting that miR‐192 could be regulating both PNPT1 and TRIP13 (Fig. 6G). Furthermore, UALCAN showed overexpression of PNPT1 in CRC tissues as compared to normal tissues indicating it to be an oncogene in CRCs (Fig. 6H). Western blot analysis of CRC cells to check the expression status of PNPT1 confirmed that PNPT1 is indeed overexpressed in HCT116^p53‐wt,MSI^, HCT116^p53‐null,MSI^, SW480^p53‐mut,MSS^, and HT29^p53‐mut,MSS^ as compared to SV‐40 transformed CRL1807 normal colon cells (Fig. 6I). Furthermore, TRIP13 knockdown has not decreased the protein levels of PNPT1 (Fig. 6J) while PNPT1 knockdown resulted in decreased levels of TRIP13 protein (Fig. 6K), suggesting that as an interacting partner, PNPT1 could be regulating TRIP13 in CRC cells.

### 3.7 Identification of TRIP13 downstream targets in CRCs

**Fig 7:**
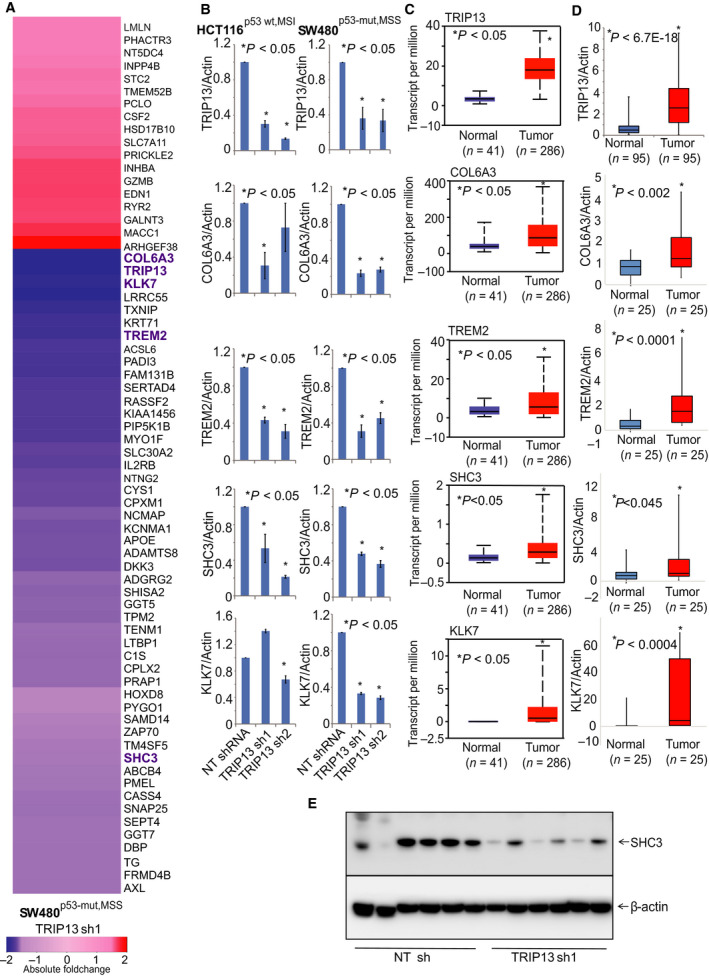
TRIP13 downstream targets in CRCs. (A) RNA sequencing data of differentially expressed genes after TRIP13 knockdown in SW480^p53‐mut,MSS^ cells as compared to control NT shRNA. (B) Validation of downstream targets of TRIP13 by performing qPCR analysis with RNA of HCT116^p53‐wt,MSI^ and SW480^p53‐mut,MSS^ cells with TRIP13 knockdown. (C) TCGA data acquired from UALCAN showing expression analysis for TRIP13 downstream targets, COL6A3, TREM2, SHC3, and KLK7. (D) qPCR analysis of COL6A3, TREM2, SHC3, and KLK7 in our cohort of CRC patients. Asterisk (*) shows statistically significant data (*P < 0.05). (E) Western blot of xenograft lysates to show the protein expression of SHC3.

To gain insight into the biological role of TRIP13 in CRCs, we used RNA sequencing to evaluate mRNA expression after TRIP13 knockdown and identified 68 differentially expressed genes, including 50 down‐regulated genes (Fig. 7A). Genes modulated by TRIP13 knockdown included COL6A3, KLK7, TREM2, and SHC3. COL6A3 [44, 45] and KLK7 [46] in CRCs, SHC3 in hepatocellular carcinomas [47], and TREM2 in gastric cancers [48] and renal cell carcinomas [49] are associated with tumor growth. We showed decreased mRNA expression of COL6A3, KLK7, TREM2, and SHC3 after TRIP13 knockdown in CRC cells (Fig. 7B). TCGA data showed that these genes were upregulated in CRCs as compared to normal tissues (Fig. 7C; Supplementary Fig. 7). With the frozen cohort, we assessed the overexpression of TRIP13 targets in CRCs and found overexpression of COL6A3, KLK7, TREM2, and SHC3 in CRCs (*n* = 25) as compared to matched normal colon tissues (*n* = 25; Fig. 7D). Western blot analysis of xenograft tissue lysates confirmed decreased expression of SHC3 after TRIP13 knockdown (Fig. 7E). These data confirmed that COL6A3, KLK7, TREM2, and SHC3 are downstream targets of TRIP13 and indicate that TRIP13 is involved in CRC progression through these molecules.

## 4 Discussion

Since, for various malignancies, including CRC, TRIP13 is associated with CIN, aneuploidy, and poor prognosis, the present study focused on identifying its downstream targets and its involvement in WNT/β‐catenin pathways and EGFR activation, which are responsible for CRC growth and metastasis. TRIP13 overexpression in CRCs correlates with expression of carcinoembryonic antigen (CEA), carbohydrate antigen 19‐9 (CA19‐9), and tumor stage (pTNM) [23]. Further, TRIP13 interacts with YWHAZ, a 14‐3‐3 protein that mediates the G2‐M transition and the EMT [23]. The current study identified additional molecular targets (COL6A3, TREM2, SHC3, and KLK7) that are regulated by TRIP13 in CRCs and showed that TRIP13 is overexpressed in CRCs irrespective of pathologic stage, patient's race/ethnicity, gender, and age. *In vitro* studies proved the functional relevance of TRIP13 in proliferation, colony formation, invasion, and migration of CRC cells regardless of their p53 and MS status. We also showed that, in CRC cells, microRNA‐192 regulates expression of TRIP13. A prominent finding was demonstration of the role of TRIP13 in experimental metastasis; inhibition of metastasis by its knockdown has not previously been reported for CRC.

We initiated the current investigation by analyzing TCGA data on expression of TRIP13 and found that, as reported for other studies [23], higher TRIP13 expression in CRCs. This result was corroborated by the analysis of gene and protein expression levels of TRIP13 in our FFPE samples (by IHC) and frozen samples (by qRT‐PCR) of CRCs compared to their matched normal samples. Our analyses showed that TRIP13 upregulation in CRCs was independent of p53 and MS status of tumor, pathologic stage, histology, and grade, race/ethnicity, gender, and age. These findings indicated that TRIP13 upregulation is a marker of CRCs that is not confounded by demographic factors, p53, or MS status. The consistent upregulation of TRIP13 in CRCs indicated that it is a potential molecular biomarker for tumor aggressiveness and that it is a target for therapy. Moreover, there was upregulated expression of TRIP13 in liver metastases of CRCs as compared to their corresponding adjacent normal liver tissues. The current findings of TRIP13 overexpression show an apparent biological significance of TRIP13 in CRC growth and metastasis.

To evaluate the oncogenic potential of TRIP13 in CRCs, we used lentiviral TRIP13 shRNA transfected into CRC cells that exhibit different p53 and MS status. Although multiple genetic alterations contribute to the development of CRCs, CRC cell lines that demonstrate wt, mutated, or null status for key molecules (e.g., *KRAS*, *BRAF*, and *PTEN*) are not available. However, well‐characterized CRC cells for p53 status (wt, null, and mut) and MS status are available. Moreover, most CRCs exhibit p53 mutations (more than 50%) and MSS (about 85% of sporadic CRCs) [50]. Further, MSS‐CRCs have a proficient mismatch repair mechanism that contributes to the aggressive behavior of CRCs [51]. In addition, we have a large series of well‐characterized CRC tissues and cells for p53 (wt, null and mut) and MS status. Since the function of TRIP13 in the background of p53 and MS status has not been studied for any other malignancy, demonstration of a functional connection between these two molecular determinants with TRIP13 is relevant for treatment of CRCs. Our findings showed that TRIP13 knockdown inhibits cell proliferation, colony formation, invasion, and cell motility, independent of the p53 and MS status of the cells. Similar results were evident in our expression profiling of CRCs for TRIP13; its high expression was independent of p53 and MS status. Consistent with these results, low expression of TRIP13 in CRC cells slowed spheroid formation, a process that mimics tumor growth, and demonstrated that TRIP13 is involved in tumor progression.

Among patients with CRC, distant metastasis is the primary cause of cancer‐related deaths [52]. CRC frequently metastasizes to liver, lungs, and peripheral lymph nodes and rarely to kidney and bone. Our study, which utilized HCT116^p53‐wt,MSI^ and HT29^p53‐mut,MSS^ CRC cells that metastasized to liver, lungs, kidney, and bone, showed the involvement of TRIP13 in distant metastasis of CRC. TRIP13 knockdown by shRNA decreased the capacity of CRC cells to spread to lungs, liver, kidney, and bone; the inhibition was independent of the p53 and MS status. In hepatocellular carcinoma (HCC) cells, TRIP13 knockdown increases levels of the epithelial marker, E‐cadherin, and decreases the mesenchymal markers, vimentin, and snail. Thus, in HCC cells, TRIP13 promotes metastasis *via* inducing the EMT [53]. Further, in bladder cancer cells, TRIP13 knockdown increases E‐cadherin and decreases N‐cadherin and Snail [54]. In the current study, we found that TRIP13 overexpression in CRC progression involves activation of EMT, as TRIP13 knockdown lowers mesenchymal phenotypes and increases epithelial characteristics. These data show that TRIP13 knockdown modulates the EMT pathway.

We showed, in CRC cells, that TRIP13 was negatively regulated by miR‐192 at the post‐transcriptional level by binding to the 3′‐UTR of TRIP13 mRNA. Additionally, we observed that miR‐192 overexpression in CRC cells lowered cell proliferation, colony formation, invasion, and spheroid‐forming capacity. However, in prostate cancer cells, miR‐515‐5p inhibits cell migration and invasion through targeting TRIP13 at its 3′‐UTR [55]. Moreover, prior studies in pancreatic cancer [56], lung cancer [57], breast cancer [58], and CRC [59] demonstrate that miR‐192 regulates expression of SERPINE1, RB‐1, BMP‐6, and DHFR and functions as a tumor suppressor. There are low levels of miR‐192 in CRCs [60]; in particular, its expression is low in advanced stages as compared to CRCs confined to the colorectum (stages I and II) [61]. Overexpression of miR‐192 impedes CRC metastasis of HCT116 cells by regulating expression of the pro‐metastatic genes Bcl2, ZEB2, and VEGFA [61]. We conclude that, in CRC cells, miR‐192, an anti‐oncogenic miRNA, regulates TRIP13 expression, suggesting that TRIP13 knockdown by miR‐192 contributes to reduced metastasis by down‐regulation of pro‐metastatic genes.

Further mechanistic investigations unraveled that PNPT1 as an interacting partner and a regulator of TRIP13 protein and in silico data analyses of CRC tissues and protein expression profiles of CRC cells have indicated PNPT1 as an oncogene in CRC. PNPT1, a 3′,5′‐exoribonuclease, is an interferon inducible gene, induces the expression of cytokines such as IL‐6, IL‐8, RANTES, and MMP3 [62]. A previous study has showed PNPT1 as a tumor‐associated antigen in CD‐40‐activated leukemic cells [63]. Findings of the current study that decreased levels of TRIP13 observed in PNPT1 knockdown CRC cells, but not down‐regulation of PNPT1 expression in TRIP13‐inhibited cells, suggest its interacting partner, PNPTI, regulates the oncogenic functions of TRIP13. Furthermore, we found that miR‐192 binding sites to PNPT1 resemble TRIP13 binding sites, indicating that miR‐192 regulates both TRIP13 and PNPT1.

Understanding tumor biology is relevant for improving therapeutic strategies. For cancers, EGFR overexpression and constitutive activation are related with a poor prognosis [64]. In the current study, we showed that, for CRC cells, less EGFR phosphorylation upon TRIP13 knockdown, suggesting that the binding of TRIP13 to EGFR is disrupted, thus resulting in less phosphorylation and leading to suppression of the EGFR signaling pathway. The suppression of EGFR phosphorylation is independent of the *RAS* mutation status, as HCT116^p53‐wt,MSI^ and SW480^p53‐mut,MSS^ cells exhibited mutations in KRAS at the G13D and G12V codons, respectively, and both cell lines showed lower EGFR phosphorylation after TRIP13 knockdown. A prior study demonstrates that TRIP13 binds EGFR and activates its signaling pathway [42]. However, the present study did not find, in CRC cells, a direct interaction between TRIP13 and EGFR. However, previous studies show activation of the EGFR pathway by FGFR4 in lung cancers [65] and colon cancers [43]. Thus, the current study investigated the interaction between TRIP13 and FGFR4 and results revealed a noticeable interaction between these two oncogenes. Moreover, we found that, in CRC cells, down‐regulation of TRIP13 led to lower EGFR and Akt phosphorylation. However, further delineation of the TRIP13‐FGFR4‐EGFR axis, including EGFR ligands, is warranted to elucidate the mechanisms of EGFR activation. Aberrant activation of the WNT/β‐catenin pathway is frequently present in CRCs [66]. Constitutive activation of Wnt signaling leads to nuclear translocation of β‐catenin and its binding to the TCF/LEF family of transcription factors [67]. The present study showed that TRIP13‐depleted CRC cells had less activity for β‐catenin as well as lower levels of LEF1 and TCF1. In HCCs, it was shown that TRIP13 regulates the WNT/β‐catenin pathway [53]. For CRCs, cyclin D1, a Wnt target gene, is transcriptionally activated by β‐catenin [68]. Further, cyclin D1, which is regulated by EGFR, may contribute to the emergence of EGFR‐driven tumorigenesis [69]. Our results showed that protein levels of cyclin D1 were lower in TRIP13‐deficient CRC cells. Thus, our conclusion is that, for CRCs, TRIP13 regulates EGFR as well as β‐catenin‐dependent expression of cyclin D1 and that it is involved in the WNT/β‐catenin pathway.

Our RNA sequencing studies with TRIP13 knockdown cells identified downstream molecular targets, including COL6A3 (collagen/extracellular matrix), TREM2 (inflammation), SHC3 (MVP/MEK/ERK activation), and KLK7 (MAPK signaling). Prior studies have implicated these TRIP13 targets in the progression of various cancers, including COL6A3 [44, 45] and KLK7 [46] in CRCs, SHC3 in hepatocellular carcinomas [47], and TREM2 in gastric cancers [48] and renal cell carcinomas [49]. Our findings were validated with human samples, which showed upregulation of COL6A3, KLK7, TREM2, and SHC3 in CRCs compared to matched normal colon tissues. Thus, in CRCs, COL6A3, TREM2, SHC3, and KLK7 are downstream targets of TRIP13.

Based on the crystal structure of TRIP13, a small molecule inhibitor, DCZ0415, was designed to target this protein [70]. As established with experimental models and with primary cells derived from drug‐resistant patients with myeloma, this compound inhibits progression of myeloma [70]. Treatment of immunocompetent models with DCZ0415 increases immune cells (CD3, CD4, and CD8) and inhibits NF‐κB activity, suggesting the immunotherapeutic value of inhibition of TRIP13 [70]. Thus, future studies should be designed to test the translational value of DCZ0415 to target TRIP13 in CRC animal models.

## 5 Conclusion

In conclusion, our work showed that expression of TRIP13 is regulated by miR‐192 and that its overexpression in CRCs is independent of tumor stage and patient race/ethnicity, age, and gender and p53 and MS status. Depletion of TRIP13 caused less cell proliferation, colony formation, cell motility, invasion, spheroid‐forming capacity, tumor growth, and distant metastasis regardless of p53 and MSI status. TRIP13 depletion also caused suppression of EGFR activation and WNT/β‐catenin signaling. Additionally, COL6A3, KLK7, TREM2, and SHC3 were identified as downstream targets of TRIP13. In sum, we conclude that TRIP13 promotes CRC metastasis and that this enzyme is a potential target for treatment of CRC.

## Author contributions

UM, SV, and SA made conceptualization; UM, SV, SA involved in methodology; SA, H‐GK, BVSKC, NG, PB, AE, SAD investigated the study; UM, MJH, SV, PKD provided the resources; MB and DSC made statistical and bioinformatics data analysis; SA, UM, SV prepared original draft; UM, SV, SA reviewed and edited the manuscript; UM supervised the study.

## Conflict of interest

The authors declare no conflict of interest.

## Supporting information


**Fig. S1.** TRIP13 mRNA expression in CRCs.
**Fig. S2.** TRIP13 protein overexpression in CRCs is independent of pathologic stage, patient's race, gender and age.
**Fig. S3.** Wound‐healing assay performed to assess migration of CRC cells.
**Fig. S4.** TRIP13 knockdown lowers CRC metastasis.
**Fig. S5.** TRIP13 knockdown decreases metastasis of HT29^p53‐mut,MSS^ cells.
**Fig. S6.** TRIP13 knockdown decreases metastasis of HCT116^p53‐wt,MSI^ cells.
**Fig. S7.** Heat‐map showing overexpression of TRIP13 downstream targets.
**Table S1.** List of shRNA sequences used in this study.
**Table S2.** List of qPCR Primer sequences used in this study.
**Table S3.** List of antibodies used in this study.
