# Supplementary material for: TRIP13 promotes metastasis of colorectal cancer regardless of p53 and microsatellite instability status
Source: Mol Oncol. 2020 Oct 28;14(12):3007–29. doi: 10.1002/1878-0261.12821 (PMC7718953; doi:10.1002/1878-0261.12821)
Supplement: Supplementary file 1 — Fig. S1. TRIP13 mRNA expression in CRCs. Fig. S2. TRIP13 protein overexpression in CRCs is independent of pathologic stage, patient's race, gender and age. Fig. S3. Wound‐healing assay performed to assess migration of CRC cells. Fig. S4. TRIP13 knockdown lowers CRC metastasis. Fig. S5. TRIP13 knockdown decreases metastasis of HT29p53‐mut,MSS cells. Fig. S6. TRIP13 knockdown decreases metastasis of HCT116p53‐wt,MSI cells. Fig. S7. Heat‐map showing overexpression of TRIP13 downstream targets. Table S1. List of shRNA sequences used in this study. Table S2. List of qPCR Primer sequences used in this study. Table S3. List of antibodies used in this study. [file MOL2-14-3007-s001.docx]

**Supplementary Material**

**TRIP13 promotes metastasis of colorectal cancer regardless of p53 and microsatellite instability status**

Sumit Agarwal^1^, Michael Behring^1^, Hyung-Gyoon Kim^1^, Darshan S. Chandrashekar^1^, Balabhadrapatruni V. S. K. Chakravarthi^1^, Nirzari Gupta^2^, Prachi Bajpai^1^, Amr Elkholy^1^, Sameer Al Diffalha^1^, Pran K Datta^3,4,5^, Martin J. Heslin^4,5^, Sooryanarayana Varambally^1,5,#^, and Upender Manne^1,4,5,#,*^

^1^Department of Pathology, University of Alabama at Birmingham

^2^Department of Chemistry, University of Alabama at Birmingham

^3^Division of Hematology and Oncology, Department of Medicine, University of Alabama at Birmingham

^4^Department of Surgery, University of Alabama at Birmingham

^5^O’Neal Comprehensive Cancer Center, University of Alabama at Birmingham, Birmingham, AL

35233, USA

^#^Share Senior Authorship

^*^Correspondence to: Upender Manne, MS, PhD, Professor and Director of Translational Anatomic Pathology, Wallace Tumor Institute, Room # 420A, University of Alabama at Birmingham, Birmingham, AL 35233, USA

Phone: (205)-934-4276, Email: upendermanne@uabmc.edu

**Running Title:** TRIP13 in colorectal cancer progression

**Disclosure of Potential Conflicts of Interest:** No potential conflicts of interest were disclosed.

**Supplementary tables, Related to Methods**

**SUPPLEMENTARY TABLES**

**Table S1.** List of shRNA sequences used in this study, related to Materials and Methods.

| **Gene name** | **Catalog No** | **Supplier** | **Sequence** |
| --- | --- | --- | --- |
| **TRIP13** | shRNA 1  shRNA 2 | System Biosciences, Mountain View, CA | GUACCGAUAUGGCCAAUUA  GCAAAUCACUGGGUUCUAC |

**Table S2.** List of qPCR Primer sequences used in this study, related to Materials and Methods.

| **Gene name** | **Forward primer** | **Reverse primer** |
| --- | --- | --- |
| ***TRIP13*** | AACTCCCCTTTCTGGCTCAT | TCAAACTGCTTGTCCACTGC |
| ***COL6A3*** | CGAAAGACGAAGGAACTTGC | TGTTTTCGTTTCCACCACAA |
| ***TREM2*** | CTGGCCTGCATCTTTCTCAT | CAGTTCACTGGGTGGATGTG |
| ***SHC3*** | CCCAGTTTGCAGGAAAAGAG | AGGTGTTTGCTGCCAGTCTT |
| ***KLK7*** | TCAAGGCCTCGAAGTCATTC | GCCTGGCTATTGAGCTTCAC |
| ***ACTB*** | GCACAGAGCCTCGCCTT | GTTGTCGACGACGAGCG |

**Table S3.** List of antibodies used in this study, related to Materials and Methods.

| **Antibody** | **Application** | **Dilution** | **Supplier** | **Cat. No.** |
| --- | --- | --- | --- | --- |
| TRIP13 | IB, IHC, IP | IB, 1:1000  IHC, 1:100  IP, 5 µg | PTG Labs, Chicago, IL | 19602-1-AP |
| Cyclin D1 | IB | IB, 1: 1,000 | Cell Signaling Technology, Danvers, MA | 2978P |
| LEF1 | IB | IB, 1:1000 | Cell Signaling Technology, Danvers, MA | 2230P |
| β –catenin | IB | IB, 1:1000 | Cell Signaling Technology, Danvers, MA | 9562 |
| EGFR-Y-1068 | IB | IB, 1:1000 | Cell Signaling Technology, Danvers, MA | 3777S |
| Total-EGFR | IB, IP | IB, 1:1000  IP, 5 µg | Cell Signaling Technology, Danvers, MA | ab131498 |
| β –actin-HRP | IB | IB, 1:20000 | PTG Labs, Chicago, IL | HRP-60008 |
| Cytokeratin 8+18 | IF | IF, 1:100 | Abcam, Cambridge, MA | ab17139 |
| Alkaline phosphatase (ALP) | IF | IF, 1:100 | Abcam, Cambridge, MA | ab65834 |
| PNPT1 | IB | IB, 1: 1,000 | Proteintech, Chicago, IL | 14487-1-AP |
| SHC3 | IB | IB, 1: 1,000 | Proteintech, Chicago, IL | 12436-1-AP |
| FGFR4 | IB, IP | IB, 1:1000  IP, 5 µg | Cell Signaling Technology, Danvers, MA | 8562 |
| p-AKT Ser 473 | IB | IB, 1:1000 | Cell Signaling Technology, Danvers, MA | 9271S |
| p-AKT Thr 308 | IB | IB, 1:1000 | Cell Signaling Technology, Danvers, MA | 13038S |
| Akt | IB | IB, 1:1000 | Cell Signaling Technology, Danvers, MA | 9272S |
| MMP2 | IB | IB, 1:1000 | Proteintech, Chicago, IL | 10373-2-AP |
| MMP9 | IB | IB, 1:1000 | Proteintech, Chicago, IL | 10375-2-AP |
| E-cadherin | IB | IB, 1:1000 | BD Pharmingen, San Diego, CA | 610181 |
| N-cadherin | IB | IB, 1:1000 | Proteintech, Chicago, IL | 22018-1-AP |
| Snail | IB | IB, 1:1000 | Cell Signaling Technology, Danvers, MA | 3879P |
| Anti-Rabbit IgG HRP | IB | IB, 1:5000 | PTG Labs, Chicago, IL | SA00001-2 |
| Anti-Mouse IgG HRP | IB | IB, 1:5000 | PTG Labs, Chicago, IL | SA00001-1 |
| Anti-Rabbit IgG Alexa Fluor 555 | IF | IF, 1:1000 | ThermoFisher Scientific, Eugene, OA | A21430 |
| Anti-mouse IgG Alexa Fluor 647 | IF | IF, 1:1000 | ThermoFisher Scientific, Eugene, OA | A32728 |

IB: Immunoblotting; IHC: Immunohistochemical; IP: Immunoprecipitation

**
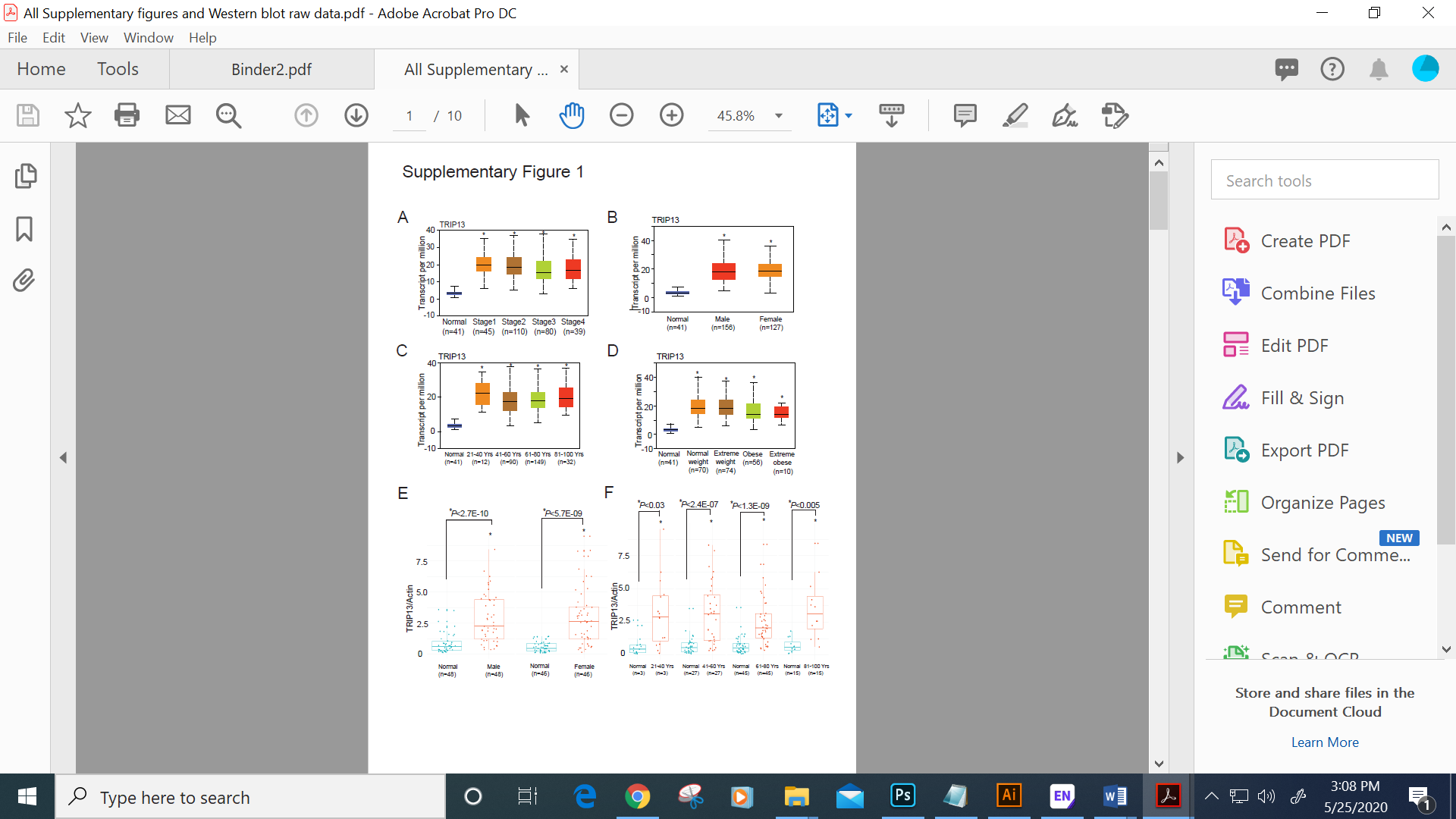
**

**Supplementary Figure S1. TRIP13 mRNA expression in CRCs.** TCGA data extracted from UALCAN showed expression with respect to (A) stages, (B) patient’s race, (C) age and (D) weight**.** Reverse transcriptase qPCR analysis of RNA expression on matched normal and CRC frozen samples showed expression according to (E) gender and (F) age. Related to Figure 1.

**
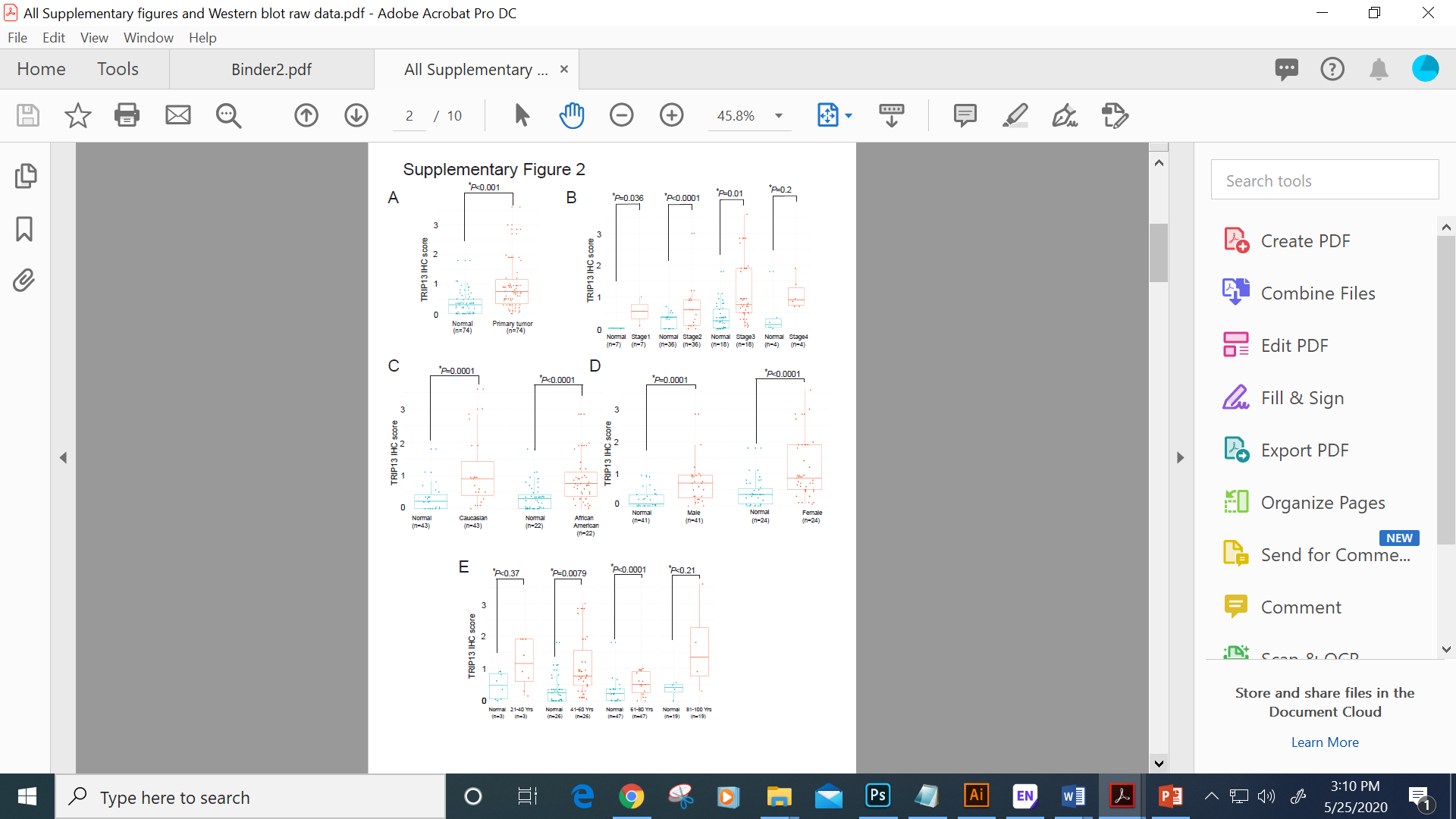
**

**Supplementary Figure S2. TRIP13 protein overexpression in CRCs is independent of pathologic stage, patient’s race, gender and age.** Plots of IHC scores of TRIP13 expression with respect to (A) CRCs and their matching normal tissues, (B) pathologic stage, (C) patient’s race, (D) gender, and (E) age. Related to Figure 2.


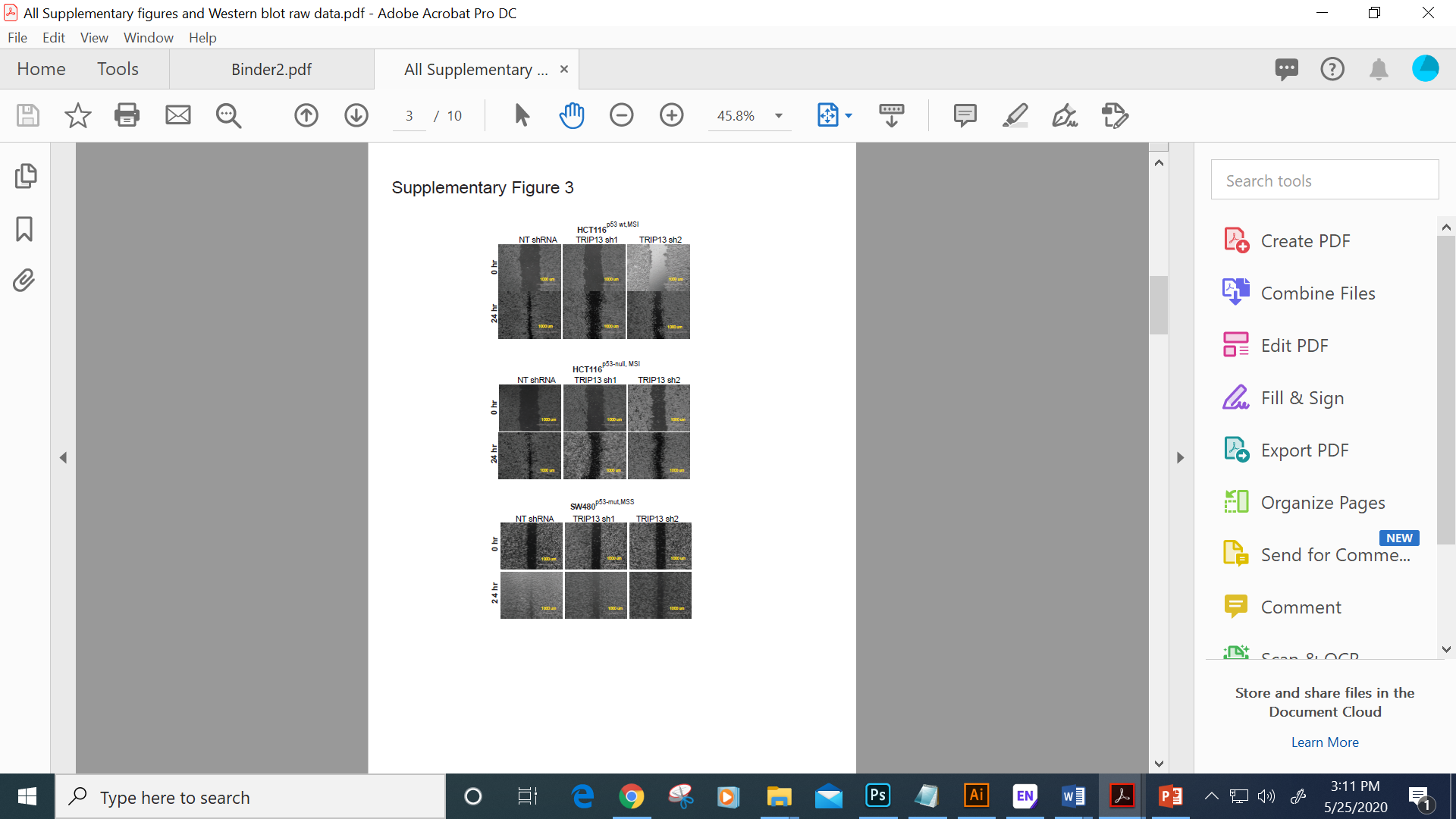


**Supplementary Figure S3.** Wound-healing assay performed to assess migration of CRC cells. At 24 hours after scratching, wound closure was determined. Related to Figure 3.

**
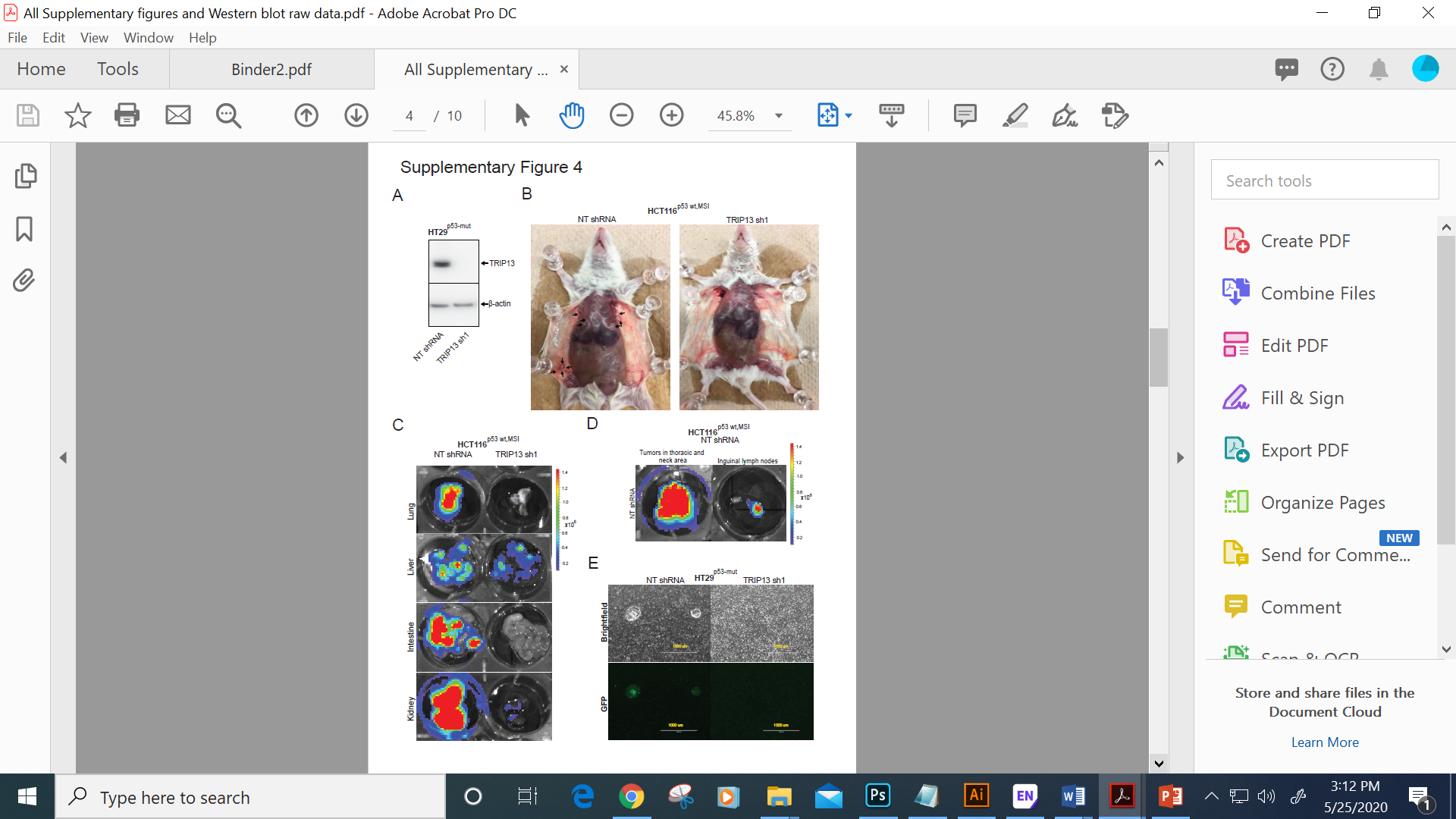
**

**Supplementary Figure S4. TRIP13 knockdown lowers CRC metastasis.** (A) Immunoblot analysis of cell lysates showing TRIP13 protein ablation in luciferase tagged HT29^p53-mut,MSS^ infected with NT shRNA or TRIP13 shRNA. β-Actin was used as a loading control. (B) Representative necropsy picture demonstrating lymph node metastasis at inguinal sites and the extent of metastasis at other areas as shown with arrows in control NT shRNA mice. (C) *Ex vivo* imaging of organs procured from mice injected with HCT116^p53-wt,MSI^ cells. (D) Control NT shRNA *ex vivo* imaging of inguinal lymph nodes and tissues obtained from the thorax and neck areas. (E) Representative phase contrast and GFP images of mice bone marrow injected with HT29^p53-mut,MSS^ after 8 days; 1000 µm. Related to Figure 4.

**
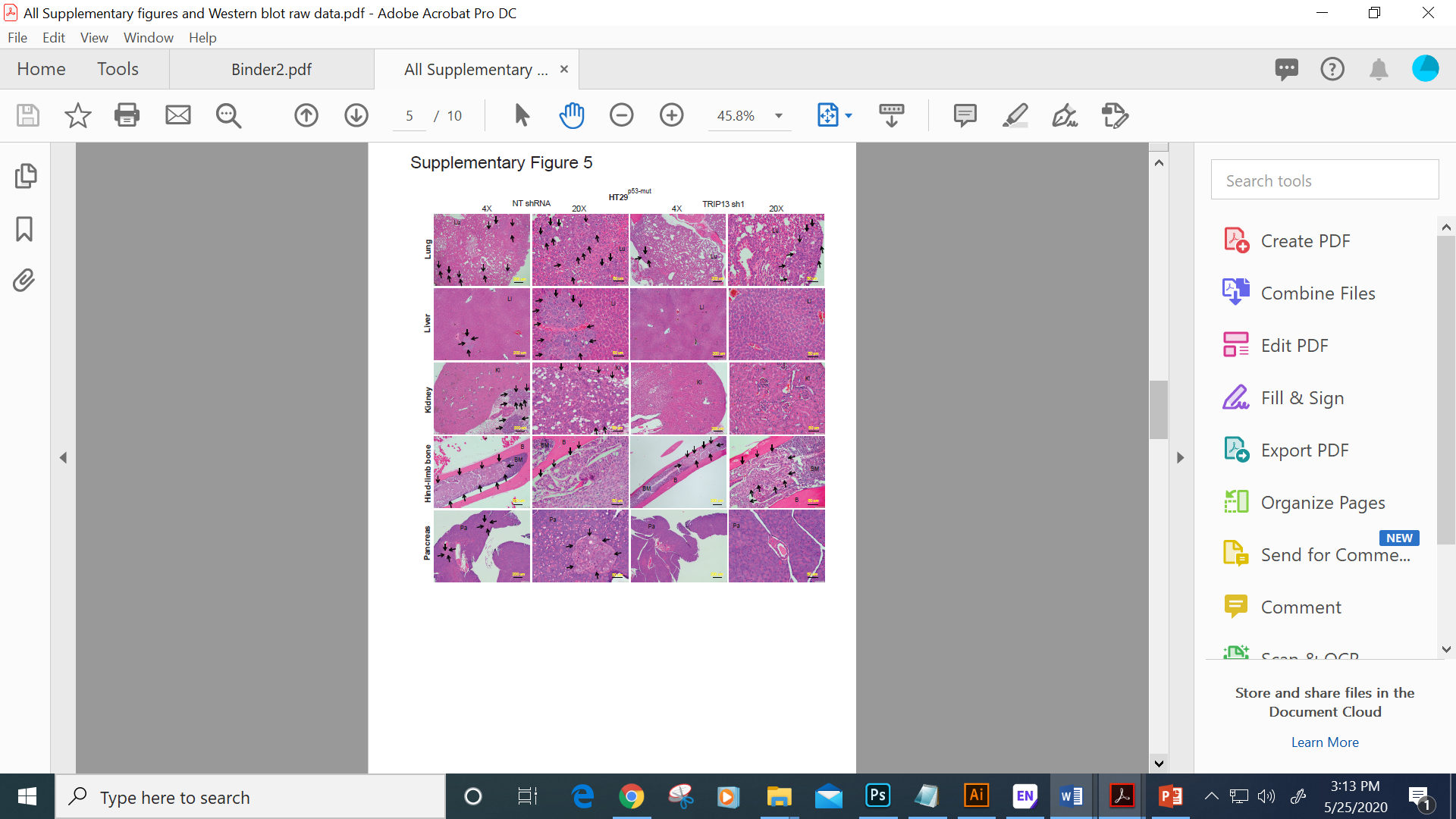
**

**Supplementary Figure S5. TRIP13 knockdown decreases metastasis of HT29^p53-mut,MSS^** **cells.** Representative H&E images of lung, liver, kidney, hind-limb bone and pancreas from HT29^p53-mut,MSS^ cells infected with control NT or TRIP13 shRNA. Arrow shows metastatic lesions; Lu- lung; Li- liver; Ki- kidney; B- bone; BM- bone marrow; Pa- Pancreas. 4X- scale bar, 200 µm; 20X- scale bar, 50 µm. Related to Figure 4.

**
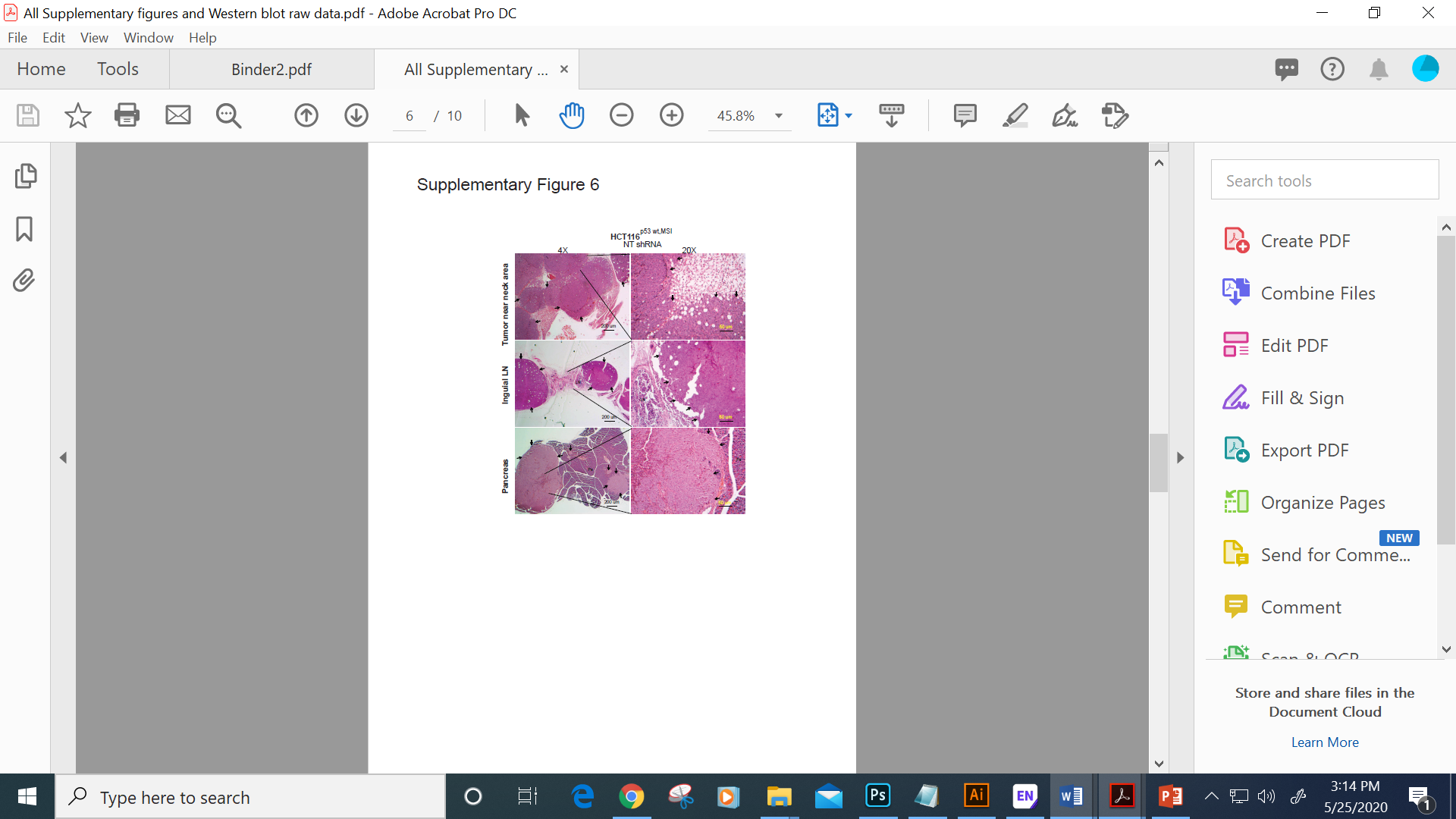
**

**Supplementary Figure S6. TRIP13 knockdown decreases metastasis of HCT116^p53-wt,MSI^** **cells.** Representative H&E images of tumors in the neck area, inguinal lymph nodes, and pancreas of mice bearing HCT116^p53-wt,MSI^ cells transfected with control NT shRNA. 4X- scale bar, 200 µm; 20X- scale bar, 50 µm. Related to Figure 4.

**
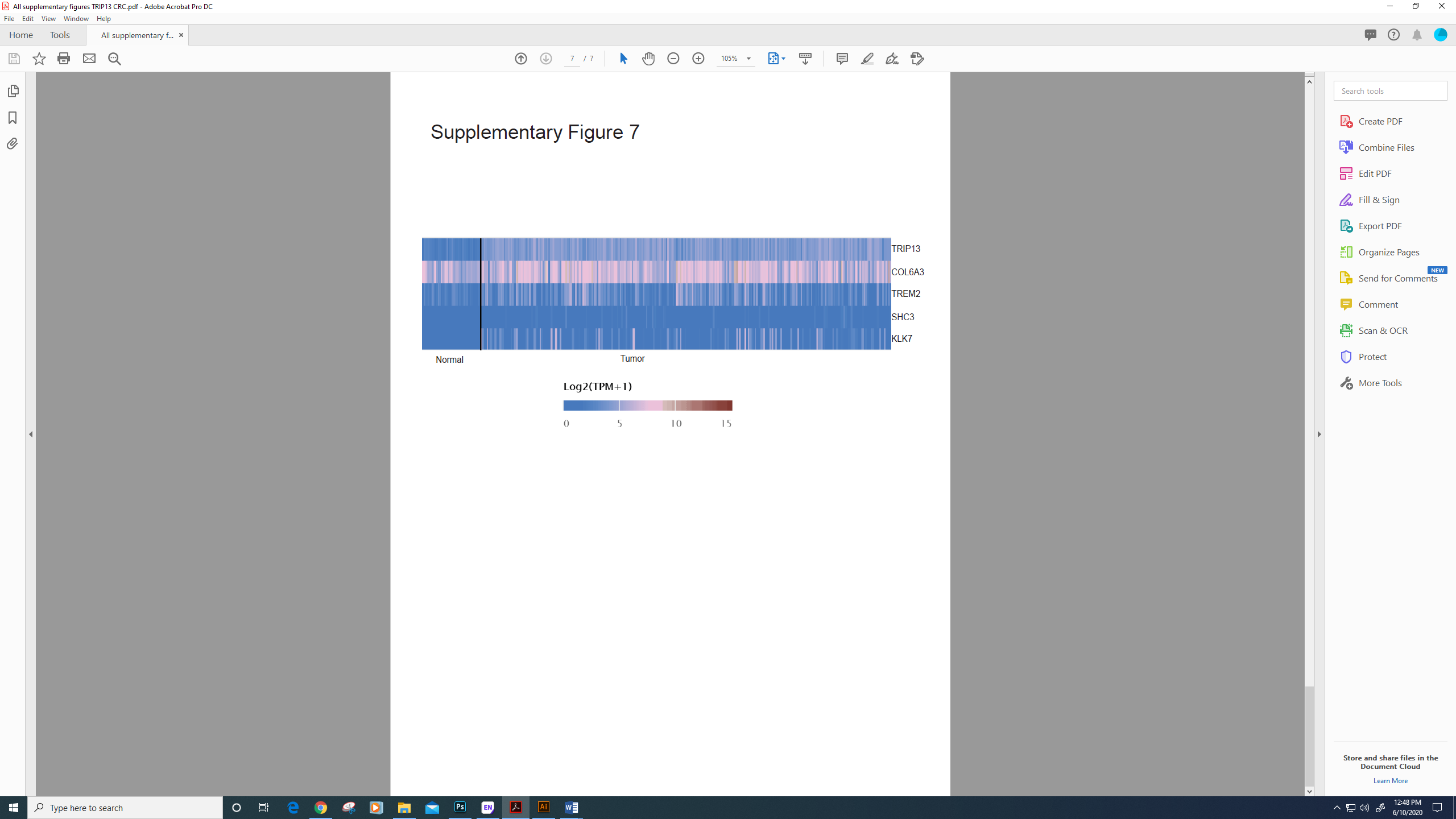
**

**Supplementary Figure S7. Heat-map showing overexpression of TRIP13 downstream targets.** Interactive heat-map plot showing RNA expression of TRIP13, COL6A3, TREM2, SHC3, and KLK7 in normal tissues and CRCs from TCGA dataset generated by web-portal UALCAN.
